# Atmospheric transport is a major pathway of microplastics to remote regions

**DOI:** 10.1038/s41467-020-17201-9

**Published:** 2020-07-14

**Authors:** N. Evangeliou, H. Grythe, Z. Klimont, C. Heyes, S. Eckhardt, S. Lopez-Aparicio, A. Stohl

**Affiliations:** 1 Norwegian Institute for Air Research (NILU), Instituttveien 18, 2007 Kjeller, Norway; 2 International Institute for Applied Systems Analysis (IIASA), 2361 Laxenburg, Austria; 3 Department of Meteorology and Geophysics, University of Vienna, UZA II, Althanstraße 14, 1090 Vienna, Austria

**Keywords:** Atmospheric science, Environmental impact, Environmental impact

## Abstract

In recent years, marine, freshwater and terrestrial pollution with microplastics has been discussed extensively, whereas atmospheric microplastic transport has been largely overlooked. Here, we present global simulations of atmospheric transport of microplastic particles produced by road traffic (TWPs – tire wear particles and BWPs – brake wear particles), a major source that can be quantified relatively well. We find a high transport efficiencies of these particles to remote regions. About 34% of the emitted coarse TWPs and 30% of the emitted coarse BWPs (100 kt yr^−1^ and 40 kt yr^−1^ respectively) were deposited in the World Ocean. These amounts are of similar magnitude as the total estimated direct and riverine transport of TWPs and fibres to the ocean (64 kt yr^−1^). We suggest that the Arctic may be a particularly sensitive receptor region, where the light-absorbing properties of TWPs and BWPs may also cause accelerated warming and melting of the cryosphere.

## Abstract (web summary)

Plastic pollution is a critical concern across diverse ecosystems, yet most research has focused on terrestrial and aquatic transport, neglecting other mechanisms. Here the authors show that atmospheric transport is a major pathway for road plastic pollution over remote regions.

## Introduction

Global annual plastic production reached 359 million tonnes in 2018^1^ and, consequently, plastic pollution in freshwater^2^, marine^3^ and terrestrial^4^ ecosystems has received a lot of attention recently. Plastics are released into the environment as macroplastic (>5 mm)^5^, microplastic (1 μm to 5 mm)^6^ and nanoplastic (<1 μm)^7^ particles that can fragment into smaller sizes via photodegradation, physical abrasion, hydrolysis and biodegradation^8^. Plastics can affect coral reefs^9^, marine^10^ and terrestrial animals^11^, as well as human health^12,13^.

An important source of plastics is road traffic emissions^14,15^. Kole et al.^14^ reported global average emissions of tyre wear particles (TWPs) of 0.81 kg year^−1^ per capita, about 6.1 million tonnes (~1.8% of total plastic production). Emissions of brake wear particles (BWPs) add another 0.5 million tonnes. TWPs and BWPs are produced via mechanical abrasion and corrosion^16,17^.

Tyres consist of a mix of elastomers such as rubber (natural and synthetic)^18^, carbon black, steel cord, fibres, and other organic and inorganic components used to improve their stability^19^; TWPs are produced by shear forces between the tread and the road pavement, generating coarse particles^20^, or by volatilization generating submicronic particles^21^. The wearing process depends on the type of tyre, road surface and vehicle characteristics, as well as on the vehicle’s state of operation^22^.

Most car braking systems consist of a disc or drum with either a pair of shoes or pads mounted in callipers. Brake linings consist of binders, fibres, fillers, frictional additives or lubricants and abrasives^23–25^. Thus, BWPs are a complicated mixture of metal and plastic. BWP emissions depend on the bulk friction material^23,26^, on the frequency and severity of braking^27^, speed, weight, condition and maintenance of the automobile^28^ and the environmental conditions^23,29,30^.

Transport of TWPs and BWPs via runoff and wash-out to marine and/or freshwater ecosystems has been studied^31,32^. However, very little is known about their dispersion in the atmosphere^33–35^ and where they are deposited, despite their health impacts in animals^9–11,36^ and humans^12,37^, possibly enhanced by adsorbed toxic organic compounds and heavy metals^38^. Greater use of plastics results in more extensive consumption of fossil fuels and, in turn, in larger emissions of greenhouse gases^39^ such as methane and ethylene^40^. Since TWPs and BWPs can be present at sizes <10 µm^41^, they can remain airborne for long periods of time; different types of microplastics have been detected already in remote areas^42–47^. Considering that they are colourful particles^48^, they also absorb light and thereby decrease the surface albedo of snow and ice accelerating melting, similar to black carbon (BC)^49^.

Here, we examine atmospheric transport and deposition of TWPs and BWPs on a global scale (see “Methods”). For simplicity we often refer to these particles jointly as road microplastics, although TWPs and BWPs are not the only microplastics that are emitted by traffic (other sources include polymer-modified bitumen used for road pavement or road marking paint). Even though TWPs and BWPs contain components other than plastics (e.g., metals), plastics are the dominant component, especially for TWPs. We also speak of microplastics^50^, since we only consider the particles of mean size 0.5–9.5 µm, which can remain airborne for long periods of time.

## Results

### Annual global emissions of road microplastics

TWP emissions were calculated using two different approaches, (a) one based on detailed information of TWP emissions in Northern Europe and extrapolation using a CO_2_ ratio method^51^, and (b) one based on the GAINS (Greenhouse gas–Air pollution Interactions and Synergies) model^52^ (see “Methods”). The two methods were compared in detail (Supplementary Fig. 1) showing that TWP emissions are very similar (0.25–32 t (tonnes) year^−1^ per grid cell); therefore, we report the geometric average. Uncertainties were calculated based on different assumptions on the airborne fraction of total emissions (see “Methods”). The use of different methods in emission calculations was, in addition, a tool to cross-validate whether the emissions are realistic or not. As seen in “Methods”, the CO_2_ ratio method is based on country statistics of returned tyres. Unfortunately, similar statistics for brake wear do not exist, and therefore, only emissions from the GAINS model were used for BWPs.

**Fig. 1:**
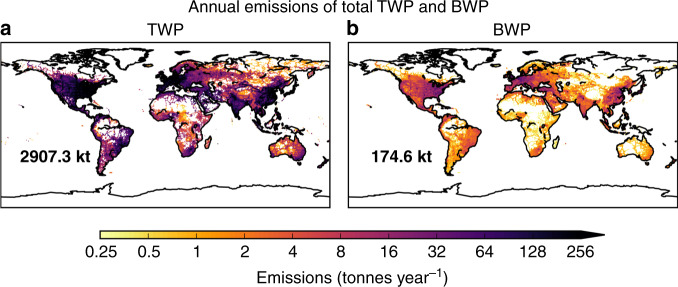
Annual gridded emissions of road microplastics. Global annual emissions of total road microplastics (tyre wear particles, TWPs, in **a**, and brake wear particles, BWPs, in **b**). TWP emissions are the average of the calculated emissions using the CO_2_ ratio method and the GAINS model. Bold numbers at the left bottom of each panel represent the annual emissions of total TWPs and BWPs from road vehicles for 2014, which were estimated to be 2907 and 174.6 kt, respectively.

Emissions of road microplastics are concentrated in the eastern US, Northern Europe and large urbanized areas of Eastern China, Middle East and Latin America where vehicle densities are highest (Fig. 1, Supplementary Fig. 1). Annual total global TWP emissions were 2907 kt (kilotonnes) year^−1^ (3434 kt year^−1^ from the CO_2_ ratio method and 2380 kt year^−1^ from the GAINS model), while BWP were 175 kt year^−1^ (Fig. 1a, b). For the particulate matter 2.5 (PM2.5) and PM10 size fractions, TWP emissions were 29 kt year^−1^ (12–75 kt year^−1^) and 288 kt year^−1^ (113–826 kt year^−1^), respectively (Table 1). The highest emissions were calculated for Asia (excluding Russia) (PM2.5 4.8–30 kt year^−1^, mean 12 kt year^−1^; PM10 85.0–167 kt year^−1^, mean 113 kt year^−1^) and North America (PM2.5 2.6–16 kt year^−1^, mean 6.4 kt year^−1^; PM10 46–90 kt year^−1^, mean 64 kt year^−1^) (Fig. 1). The annual global emissions of BWP were 98.2 kt year^−1^ (63.4–152 kt year^−1^) for PM2.5 and 146 kt year^−1^ (85.8–248 kt year^−1^) for PM10 (Fig. 1, Supplementary Fig. 2). BWP emissions were very similar in Europe and North America, for both PM2.5, but highest in Asia (Table 1, Supplementary Fig. 2).

**Table 1:** Annual road microplastic emissions.

	Europe	Asia	Russia	North America	Central America	South America	Africa	Oceania	Total
PM2.5 TWP	2.3–15 (5.8)	4.8–30 (12)	0.26–1.6 (0.64)	2.6–16 (6.4)	0.32–2.0 (0.80)	0.8–5.0 (2.0)	0.56–3.5 (1.4)	0.19–1.2 (0.48)	12–75 (29)
PM10 TWP	42–82 (58)	85.0–167 (113)	4.6–9.0 (6.4)	46–90 (64)	5.7–11 (8.0)	14–28 (20)	10–20 (14)	3.4–6.8 (4.8)	113–826 (288)
PM2.5 BWP	13–32 (21)	26–62 (40)	2.5–6.0 (3.9)	11–26 (17)	1.7–4.0 (2.6)	4.4–10 (6.8)	3.4–8.1 (5.2)	0.97–2.3 (1.5)	63.4–152 (98.2)
PM10 BWP	28–37 (32)	50–67 (58)	6.9–9.1 (7.9)	22–29 (25)	3.2–4.2 (3.7)	8.4–11 (9.7)	6.5–8.6 (7.5)	1.9–2.5 (2.2)	85.8–248 (146)

Annual continental emissions (in kt) of road microplastics (tyre wear particles, TWPs, and brake wear particles, BWPs) in PM2.5 and PM10 size modes averaged for the two different methodologies used (CO_2_ ratio and GAINS model emissions). Corresponding ranges are variations of continental geometric standard deviations from geometric means (presented within parentheses) following a log-normal distribution (see “Methods”). The airborne PM10 fraction was assumed to be 2.5, 5, 10, 20 and 40% of the total TWP emissions, while the PM2.5 was assumed to be 0.25, 0.5, 1, 2 and 4% of the total TWP emissions. For BWPs, it was assumed that 30, 40, 50, 60 and 70% of total BWPs are PM2.5 and 60, 70, 80, 90 and 100% of the total BWPs are PM10. Note that Russia has been excluded from both Europe and Asia and is listed separately, while America has been divided into three parts (north, central and south).

### Atmospheric transport and deposition of road microplastics

Surface concentrations of TWPs range between a few ng m^−3^ and 20 ng m^−3^ for PM2.5 and up to 50 ng m^−3^ for PM10 (Supplementary Movie 1). BWP surface concentrations reach 50 ng m^−3^ at maximum (Supplementary Movie 1). The highest concentrations were calculated for eastern USA, Europe and South-eastern Asia. All concentrations (TWPs 0.4 μg m^−3^ for PM2.5, 1.8 μg m^−3^ for PM10; BWPs 0.8 μg m^−3^ for PM2.5, 1.4 μg m^−3^ for PM10) were far below air quality limits for PM (annual mean 10 μg m^−3^ for PM2.5, double for PM10^53^) and lower than typical BC concentrations in remote regions^54^. The annual mean modelled lifetime of PM2.5 TWPs was estimated to be 28 ± 2.7 days (range 18–37 days), while for PM10 it was equal to 8.3 ± 1.0 days (range 5.5–11 days). Accordingly, for BWPs, the annual mean modelled lifetime was calculated as 28 ± 2.8 days (range 17–37 days) for the PM2.5 size class and 1.3 ± 0.16 days (range 0.94–1.6 days) for PM10. The large calculated lifetimes for PM2.5 road microplastics are due to the small scavenging coefficients for in-cloud used in the model, as it was assumed that road microplastics should be rather hydrophobic (Methods). For comparison, a typical lifetime for atmospheric black carbon in the PM2.5 size mode is 3–11 days^49^.

**Fig. 2:**
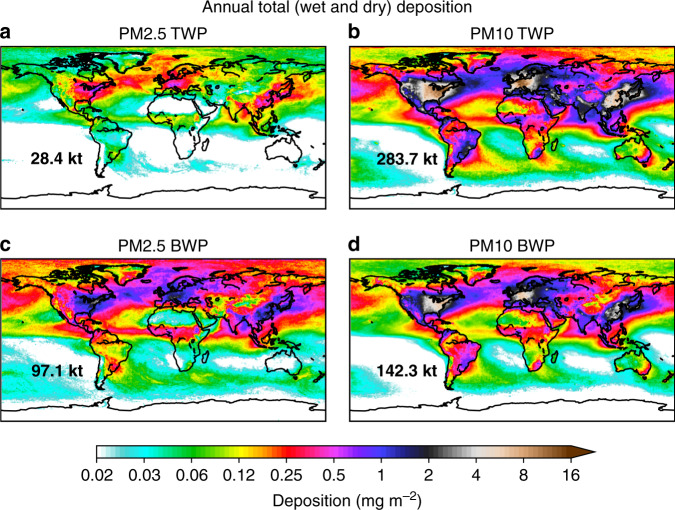
Wet and dry deposition of road microplastics. Annual total (wet and dry) deposition of tyre wear particles (TWPs) and brake wear particles (BWPs) in PM2.5 and PM10 size classes, respectively. The projected deposition has been calculated as the geometric mean of all simulations using TWP emissions estimated using the CO_2_ ratio method and the GAINS model and using BWP emissions calculated from the GAINS model, respectively. The simulations comprise 120 ensemble members with different assumption for the airborne fraction (five for each of the PM2.5 and PM10 fractions), particle size distribution (eight for each of the PM2.5 and PM10 fractions) and CCN/IN (cloud condensation nuclei/ice nuclei) efficiency (three different sets of scavenging coefficients per fraction) following a log-normal distribution (see “Methods”). Bold numbers at the left bottom of each panel represent the annual total deposition of TWPs and BWPs from road vehicles in PM2.5 and PM10 sizes for year 2014.

Annual deposition maps (Fig. 2) show that smaller road microplastic particles (PM2.5) are dispersed more widely than larger ones (PM10). PM10 road microplastics are deposited mainly close to the hotspot emission regions (North America, Europe and South-eastern Asia) (Fig. 2). Of the annual global TWP PM2.5 emission of 29 kt year^−1^ (12–75 kt year^−1^), ~1.7 kt year^−1^ (0.60–4.8 kt year^−1^) were deposited over Europe, 4.3 kt year^−1^ (1.5–12 kt year^−1^) over Asia, 3.3 kt year^−1^ (1.1–9.6 kt year^−1^) in America, and much lower amounts in Africa and Oceania (<4% of the total deposited mass). Overall, ~43% (4.3–34 kt year^−1^, mean 12 kt year^−1^) of the total deposited mass of PM2.5 TWPs was deposited on land and ~57% (5.3–48 kt year^−1^, mean 16 kt year^−1^) in the ocean (Table 2). About 8.1 kt year^−1^ (2.8–23 kt year^−1^) of PM2.5 TWPs were deposited on ice and snow surfaces (polar regions, mountains, etc.). Accordingly, annual total deposition of PM10 TWPs was 284 kt year^−1^ (102–787 kt year^−1^) with ~65% (68.1–497 kt year^−1^, mean 184 kt year^−1^) deposited on land. Around 28 kt year^−1^ (10–76 kt year^−1^) of the TWPs were deposited on snow and ice. The vast majority (~60%) was deposited in Europe, America, Russia and Asia (Table 2). Although deposition of TWPs to Antarctica was very small compared to other regions (0.03 kt year^−1^ for PM2.5, 0.01 kt year^−1^ for PM10), other forms of microplastics have been determined there likely transported via sea and/or air^55^.

**Table 2:** Annual deposition of road microplastics.

	Europe	Asia	Russia	North America	Central America	South America	Africa	Oceania	Antarctica	Land	Ocean	Snow/ice	Total
PM2.5 TWP	0.60–4.8 (1.7)	1.5–12 (4.3)	0.53–3.6 (1.5)	0.76–6.4 (2.2)	0.11–0.90 (0.32)	0.27–2.3 (0.80)	0.38–3.2 (1.1)	0.03–0.2 (0.08)	– (0)	4.3–34 (12)	5.3–48 (16)	2.8–23 (8.1)	9.6–82 (28)
PM10 TWP	11–84 (31)	24.7–180 (66.8)	4.4–32 (12)	15.5–113 (41.9)	1.9–14 (5.1)	5.1–37 (14)	4.4–32 (12)	0.70–5.1 (1.9)	– (0)	68.1–497 (184)	34.4–290 (100)	10–76 (28)	102–787 (284)
PM2.5 BWP	4.7–7.9 (6.1)	11–18 (14)	6.0–8.6 (7.2)	5.9–13 (8.8)	0.80–1.8 (1.2)	1.9–4.4 (2.9)	2.6–6.7 (4.2)	0.18–0.40 (0.27)	– (0)	30–68 (45)	29–94 (52)	20–45 (30)	59.2–162 (97.2)
PM10 BWP	13–22 (17)	27–46 (35)	6.7–11 (8.7)	18–30 (23)	1.8–3.0 (2.3)	5.6–9.5 (7.3)	5.4–9.1 (7.0)	0.92–1.6 (1.2)	– (0)	78.4–133 (102)	23–68 (40)	11–36 (20)	101–201 (142)

Annual global (wet and dry) deposition (in kt) of road microplastics (tyre wear particles, TWPs, and brake wear particles, BWPs) in PM2.5 and PM10 size bins estimated with FLEXPART version 10.4 model. TWP deposition (average values are presented within parentheses) is the geometric mean of the two simulations with emissions calculated with the CO_2_ ratio method and the GAINS model (IIASA) each including 120 ensemble members with different assumption for the airborne fraction (five for each of the PM2.5 and PM10 fractions), particle size distribution (eight for each of the PM2.5 and PM10 fractions) and CCN/IN (cloud condensation nuclei/ice nuclei) efficiency (three different sets of scavenging coefficients per fraction). BWP deposition was calculated in the same way (120 ensemble members), but only using emissions from the GAINS model. Uncertainties of TWP and BWP deposition are expressed with the geometric standard deviation taking into account all the simulations (120). The results are given in ranges based on the variation of the geometric standard deviation from the geometric mean (see “Methods”).

Of the 97 kt year^−1^ (59.2–162 kt year^−1^) annual total deposition of PM2.5 BWPs, 45 kt year^−1^ (30–68 kt year^−1^) were deposited on land (~46%) and 52 kt year^−1^ (29–94 kt year^−1^) in the World Ocean (~54%) (Table 2). About 14 kt year^−1^ (11–18 kt year^−1^) were deposited in Asia, 12.9 kt year^−1^ (8.6–19 kt year^−1^) in America, 6.1 kt year^−1^ (4.7–7.9 kt year^−1^) over Europe and 7.2 kt year^−1^ (6.0–8.6 kt year^−1^) in Russia. A significant amount (~31%) of 30 kt year^−1^ (20–45 kt year^−1^) was deposited on snow and ice surfaces. As regards to PM10 BWPs, half of the deposition (~53%) occurred in Asia, Europe and North America. About 72% (78.4–133 kt year^−1^, mean 102 kt year^−1^) were deposited on the land and the rest in the ocean, and only 20 kt year^−1^ (11–36 kt year^−1^) on snow and ice surfaces (~14% of global deposited mass). Similar to TWPs, transport to Antarctica was small compared to other continents (0.04 kt year^−1^ for PM2.5, 0.01 kt year^−1^ for PM10). The slightly smaller relative deposition of BWPs over the ocean compared to TWPs in both particle sizes (Table 2) can be attributed to the higher particle density of BWPs (see “Methods”), which leads to more rapid deposition.

### Road microplastics in snow-covered land and ice surfaces

**Fig. 3:**
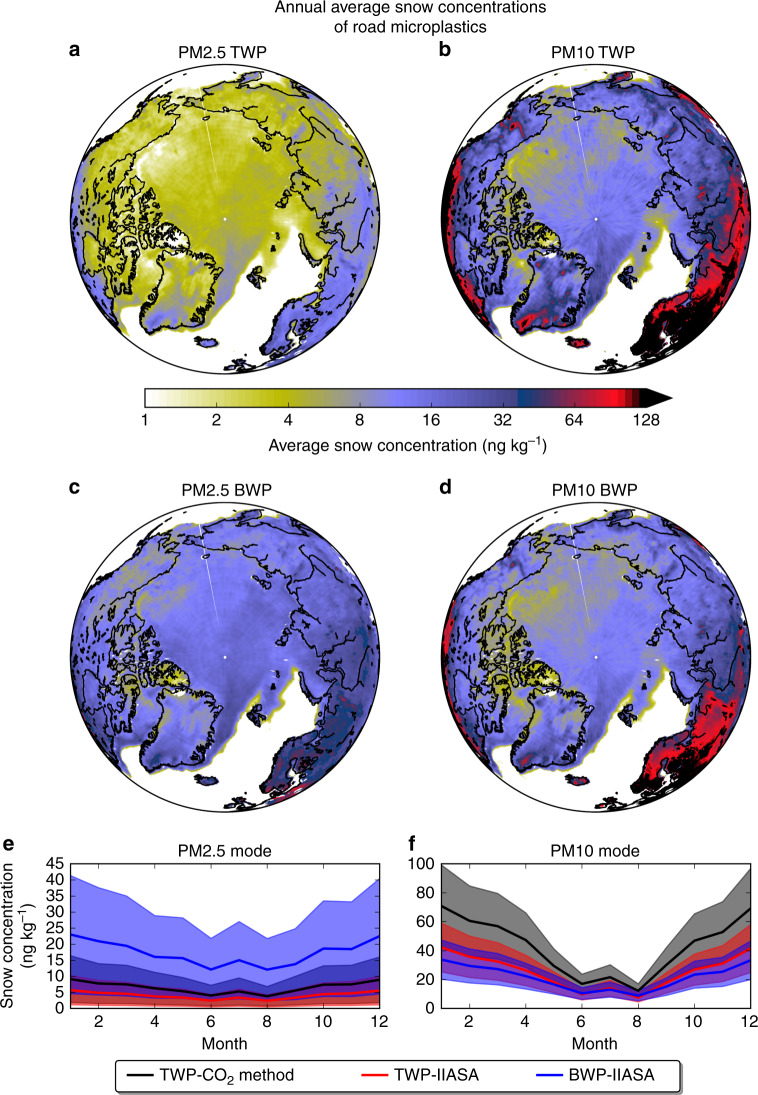
Road microplastics in snow and ice. Annual average concentrations of road microplastics in Arctic snow (**a**–**d**) in ng kg^−1^. Snow concentrations were calculated using daily fields of sea-ice area fraction and total snowfall from European Centre for Medium-Range Weather Forecast (ECMWF) combined with daily modelled deposition. The latter includes results from 120 simulations that accounted for different airborne fractions (five members for each of the PM2.5 and PM10 fractions), particle size distribution (eight members for each of the PM2.5 and PM10 fractions) and CCN/IN (cloud condensation nuclei/ice nuclei) efficiency (three different sets of scavenging coefficients per fraction) following a log-normal distribution (see “Methods”). Monthly variation of concentrations of road microplastics in the Arctic snow in both sizes (PM2.5 and PM10) are presented in (**e**, **f**). For the latter, model results using emissions from both methods are presented. Tyre wear particles (TWPs) and brake wear particles (BWPs) uncertainties have been calculated as the geometric standard deviation of all the 120 simulations with different assumption (airborne fraction, size distribution and CCN/IN efficiency, see “Methods”). Note that the smallest concentrations occur in mid-summer (**e**, **f**).

TWP concentrations in the Arctic snow ranged between 1 and 10 ng kg^−1^ of snow for PM2.5 and between 4 and 80 ng kg^−1^ for PM10 (Fig. 3). Modelled concentrations of BWPs were 2–30 ng kg^−1^ for PM2.5 and 2–70 ng kg^−1^ for PM10 (Fig. 3). For comparison, note that these values are almost three orders of magnitude lower than those of BC in Arctic snow^56,57^. It is seen that Northern Europe (e.g. Scandinavia), on one side, and North America, on the other, present higher TWP/BWP snow concentrations than the Arctic. This is a combination of the proximity to source regions and the fact that the calculation was performed only for pixels with substantial snowfall as compared to total precipitation (Supplementary Movie 2). The largest Arctic snow concentrations were predicted on the sea ice between Northern Greenland and Europe. This area receives road microplastics emitted both in North America and Europe (Supplementary Movies 3 and 5). Transport of microplastics into the Arctic occurs particularly in winter and spring (Fig. 3e, f, Supplementary Fig. 3) and is likely enhanced during positive phases of the North Atlantic Oscillation (NAO)^58^. It is known from previous work^58^ that pollution transport from North America and Europe is enhanced during positive phases of the NAO and that this effect is strongest in winter and spring. Since 1990, there have been 7 years with high NAO indexes, a negative phase around 2010 and again positive phase between 2014 and 2019^59^. Another hotspot region, in terms of snow concentrations, is Northern Eurasia (Fig. 3). This region is affected by air transport from high-emission regions further south (Supplementary Movies 3 and 5).

The uncertainty of road microplastics deposition was calculated from 120 model ensemble members, each comprising different size distribution characteristics, different coefficients for in-cloud and below-cloud scavenging and variable airborne fraction with respect to total emissions (see “Methods”). The uncertainty was calculated as the geometric standard deviation of deposition resulting from all ensemble members (see “Methods”, Supplementary Fig. 4, Supplementary Table 1 and Supplementary Table 2).

**Fig. 4:**
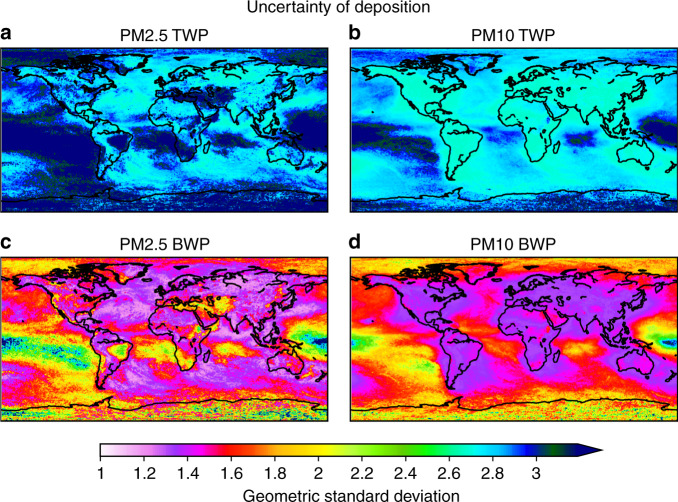
Model uncertainty. Calculated model uncertainties of deposition. Uncertainties were calculated from a model ensemble of 120 members for each of the PM2.5 and PM10 sizes, both for tyre wear particles (TWPs) and brake wear particles (BWPs). The ensemble accounts for (**a**) airborne PM10 TWP fraction to be 2.5, 5, 10, 20 and 40% of the total TWP emissions, (**b**) PM2.5 TWP to be 0.25, 0.5, 1, 2 and 4% of the total TWP emissions, while (**c**) PM2.5 BWP fraction was assumed to be 30, 40, 50, 60 and 70% of total BWPs and (**d**) PM10 BWP fraction of 60, 70, 80, 90 and 100% of the total BWPs, different wet scavenging coefficients that define CCN/IN (cloud condensation nuclei/ice nuclei) efficiency, and different assumptions on the airborne fraction in the emissions (see “Methods”). Uncertainties are given as the geometric standard deviations, since sensitivity scenarios followed a largely log-normal distribution (see “Methods”).

The relative uncertainties in deposition both for PM2.5 and PM10 road microplastics are high, with a geometric standard deviation of up to 3. This is mainly due to the great uncertainty in the size distributions of emitted TWPs and BWPs, which controls the fraction of the total mass that can become airborne and the removal properties within that fraction. Dannis^60^ found that the mean particle diameter of TWPs decreases, while vehicle speed increases, which may contribute to the large differences in reported size distributions. In an effort to explain the size distribution, Cadle and Williams^61^ suggested that the formation of sub-micron TWPs may be due to the thermal degradation of tyre polymer, with the larger particle mode being generated by mechanical abrasion. Deposition of PM2.5 road microplastics is more uncertain closer to the highest emitting continents (North-eastern USA, South-eastern China and Europe) and in tropical regions where precipitation is intense (Fig. 4a, c, e). On the contrary, the highest uncertainties for road microplastic deposition of PM10 occur in remote regions (Fig. 4b, d, e). This is related to the large sensitivity of long-range transport efficiency to gravitational settling and below-cloud scavenging of larger particles, which is relatively more important for PM10 than PM2.5.

## Discussion

To examine the susceptibility of different remote regions (e.g. oceans, Arctic, etc.) to TWP and BWP emissions, we computed the probability of road microplastics emitted globally to reach these remote regions via the atmosphere. We define the transport efficiency as the ratio between the mass of microplastics deposited in a remote area and the total mass of microplastics emitted globally. We calculate these efficiencies by masking several geographical regions (Table 3).

**Table 3:** Annual transport efficiencies of road microplastics.

	Arctic	Alps	Himalayas	Greenland	Atlantic Ocean	Pacific Ocean	Indian Ocean	Southern Ocean	Arctic Ocean	Mediterranean Sea	Baltic Sea	South China Sea
PM2.5 TWP	1.2–10 (3.5)	0.11–0.84 (0.30)	0.048–0.45 (0.15)	1.2–9.5 (3.4)	5.4–42 (15)	6.3–57 (19)	1.9–15 (5.3)	0.43–3.4 (1.2)	5.0–39 (14)	0.080–0.72 (0.24)	0.018–0.14 (0.051)	0.19–1.4 (0.52)
PM10 TWP	0.46–3.1 (1.2)	0.22–1.4 (0.56)	0.046–0.31 (0.12)	0.65–4.4 (1.7)	3.7–27 (10)	4.3–34 (12)	1.3–11 (3.9)	0.52–3.8 (1.4)	2.4–19 (6.8)	0.16–1.1 (0.42)	0.024–0.15 (0.061)	0.32–2.0 (0.78)
PM2.5 BWP	2.4–5.4 (3.6)	0.22–0.49 (0.33)	0.078–0.25 (0.14)	2.3–5.1 (3.4)	10–22 (15)	9.1–36 (18)	3.2–8.1 (5.1)	0.60–2.0 (1.1)	10–20 (14)	0.13–0.48 (0.25)	0.041–0.069 (0.053)	0.41–0.59 (0.49)
PM10 BWP	1.0–1.7 (1.3)	0.50–0.85 (0.65)	0.078–0.15 (0.11)	1.4–3.7 (2.3)	6.9–18 (11)	5.2–23 (11)	1.2–5.1 (3.2)	0.27–0.94 (0.52)	2.7–6.7 (4.3)	0.35–0.58 (0.45)	0.055–0.092 (0.071)	0.65–1.3 (0.91)

Transport efficiencies (%) of road microplastics over remote areas. Transport efficiency is defined as the ratio between the mass of microplastics deposited in a remote region divided by the total mass of microplastics emitted globally (see “Discussion”). Results are given as geometric means (presented within parentheses) that are based on simulations from different scenarios for the airborne fraction in the emissions (five for each of the PM2.5 and PM10 fractions), different particle size distribution for transport (eight for each of the PM2.5 and PM10 fractions) and different CCN/IN (cloud condensation nuclei/ice nuclei) efficiency for deposition (three different sets of scavenging coefficients per fraction). Uncertainties have been calculated as the geometric standard deviation of all simulations (120) using the aforementioned assumptions and they are expressed as ranges of the geometric standard deviation from the geometric mean (see “Methods”).

About 15% of the PM2.5 road microplastics were transported to the Atlantic Ocean (Table 3), whereas coarse particles were less efficiently deposited there (TWPs 10%, BWPs 11%). Due to the smaller size of PM2.5, their transport efficiency to the Pacific Ocean was even more strongly enhanced relative to PM10 deposition (Table 3). The South China Sea receives ~2% of airborne road microplastics, at maximum, a large amount considering its relatively small surface. This is due to the fact that South-eastern Asian emissions of microplastics tend to travel towards the South China Sea and the Western Pacific before they turn to the north, all the way to the Arctic (Supplementary Movies 3 and 5). The calculated transport of PM10 road microplastics shows a relatively high efficiency over Greenland (TWPs 1.7%, BWPs 2.3%) and over the Arctic Ocean (TWPs 6.8%, BWPs 4.3%) and much smaller to the Southern Ocean (TWPs 1.4%, BWPs 0.5%). Negligible transport to Antarctica was simulated.

A notable point here is the fact that in areas surrounded by road microplastic emissions sources, PM10 particles are more efficiently deposited than PM2.5 particles. For instance, in the Alps, the Mediterranean, Baltic and South China Seas, transport efficiencies of coarse particles were up to twice of those of the fine particles. Short exposure to PM10 particles has been highlighted as a major cause of respiratory diseases (e.g. asthma), especially considering that such regions are heavily populated^53^. The opposite is the case in remote areas that are far from emission sources such as the Arctic and Greenland, where deposition of fine particles is greater than that of coarse ones (Table 3).

Another important receptor region of global microplastic emissions is the Arctic (Table 3). It is well known that aerosols can be transported efficiently to the Arctic, in particular during the Arctic Haze season in late winter and spring^62^. We find a transport efficiency of almost 3.6% for the Arctic (excluding Greenland) and a similar transport efficiency for Greenland. These transport patterns may potentially intensify the climatic risk of microplastic pollution with respect to their ability to decrease the albedo in the Arctic and enhance snow and ice melting. In addition, road microplastics may concentrate in Arctic melt pools, with unknown ecological consequences. TWPs and BWPs constitute ~1.8% of total plastic production^14^, hence the anticipated impact of all microplastics arriving to snow and ice surfaces might be greater.

One aspect that is missing from our simulations is potential re-suspension of road microplastics. Strong winds may re-mobilize deposited microplastic particles both from the land and the ocean surface, allowing secondary transport of these particles and thus enhancing efficiency of airborne transport to remote areas such as the Arctic, somewhat similar to the well-known grasshopper effect of persistent organic pollutants^63^. Another important aspect is the fact that emissions from non-road vehicles (tractors, mining trucks and equipment, construction and forestry machinery, and even military) have not been included in our emission inventories. While these vehicles are fewer, they work in difficult conditions, are heavier and carry heavy loads leading to enhanced tyre and brake wear.

There is a lack of measurement data that could be used to validate our results. However, Bergmann et al.^43^ reported that the mean number concentration of plastic fibres detected in snow from Svalbard were 1.38 ± 1.10 ml^−1^, while in Bavaria they were 1.43 ± 0.32 ml^−1^. Most particles identified were between 11 and 25 μm, while those <11 μm were not identified due to methodological limitations. According to Stylios^64^, a microfibre is a fibre with <1 decitex (dtex) per filament, with the most common types being from polyesters and polyamides (1 dtex = 1 mg per 10 m). Since the majority of the fibres was <25 μm size, these number concentrations can be converted to mass concentrations of 3.4 ± 2.8 and 3.6 ± 0.80 ng g^−1^ in Svalbard and Bavaria, respectively. Materić et al.^65^ reported PET (polyethylene terephthalate), PPC (polypropylene carbonate) and PVC (polyvinyl chloride) concentrations in Alpine snow of 5.6–23, 11–16 and 6.9 ± 0.2 ng g^−1^, respectively. These concentrations are ~100 times higher than those estimated here for TWPs and BWPs in snow (Fig. 3), which is likely realistic considering the larger usage of these polymers as compared to TWPs and BWPs. A measurement strategy for both atmospheric concentration and deposition (e.g. in snow samples) measurements is highly recommended at different distances to major source regions in order to facilitate validation of our model results.

We calculated that out of 102–787 kt (mean 284 kt) of PM10 TWPs emitted, 34.4–290 kt (mean 100 kt) were deposited in the World Ocean. In the most recent study on riverine transport from land to ocean, van Wijnen et al.^66^ reported a total annual global export of microplastics to the ocean of 47 kt, 80% (37.6 kt) of which was produced by macroplastic degradation, and 20% (9.4 kt) from direct discharges of TWP and laundry fibres. The total annual releases of TWP in their model were assumed to be 426 kt (Table 3 in van Wijnen et al.^66^). If we assume that all microplastics are transported from land to the World Ocean over time (wash-out and runoff processes) and scale the van Wijnen et al.^66^ TWP emissions to match ours (2907 kt, Fig. 1), we calculate that 64 kt of TWP may be washed out from the land in a year. This suggests that direct deposition of airborne road microplastics is likely the most important source for the ocean.

## Methods

### TWP emission calculations based on a CO_2_ ratio method

Top-down estimates of total annual tyre wear emissions of 5700 10,000 and 100,000 tonnes, respectively, have been reported for Norway^67^, Sweden^68^ and Germany^69^ based on measurements of lifetime weight loss of returned tyres. For the rest of the globe, we did not have access to such data. To obtain global emissions (Fig. 1), we assumed a constant ratio of TWP emissions to CO_2_ emissions from the road transport sector (0.49 mg TWP g^−1^ CO_2_), using CO_2_ emissions from the CMIP6 (Coupled Model Intercomparison Project phase 6)^70^ inventory (0.5° × 0.5° resolution) for the year 2014. The TWP/CO_2_ emission ratio is the average value of the ratios obtained for Norway, Sweden, and Germany, which were all very similar: 0.43, 0.50 and 0.55 mg TWP g^−1^ CO_2_, respectively.

While the total TWP emission is relatively well constrained, the fraction of total TWP and BWP emissions that becomes airborne, assumed to be particles <10 µm (PM10), is highly uncertain. Values reported in the literature range from ~1 to 40%, while those for the PM2.5 fraction (particles <2.5 µm) are ~1%^23,30,32,41,71–74^ (Table 6 in Grigoratos and Martini^75^). We examined how sensitive the calculated concentrations of TWP are with respect to this fraction. For that, we created five scenarios that assumed that 2.5, 5, 10, 20 and 40% of the total TWP emitted are PM10 and 0.25, 0.5, 1, 2 and 4% are PM2.5 (Supplementary Table 1). We report TWP emissions as a range (geometric mean and geometric standard deviation) based on the derived emissions from the five ensemble members, each one with a different assumed fraction for the PM2.5 and PM10 mode.

### TWP and BWP emission calculations with the GAINS model

The GAINS (http://gains.iiasa.ac.at) model^76^ is an integrated assessment model where emissions of air pollutants and Kyoto gases are estimated for nearly 200 regions globally considering key economic activities, environmental regulation policies and regionally specific emission factors. For emissions of PM, GAINS provides size speciated PM discriminating PM1, PM2.5, PM10 and total PM, as well as carbonaceous particles (BC, organic carbon); detailed description of the methodology can be found in Klimont et al.^52^. Emissions of non-exhaust PM in GAINS include TWPs, BWPs and road abrasion, and the calculation is based on region-specific data and estimates of distance-driven (km vehicle type^−1^ year^−1^) and vehicle-type-specific emission rates (mg km^−1^). Distinguished vehicle types for road transport include motorcycles, cars, light duty vehicles, buses and heavy-duty vehicles. The estimates of distance driven for 2015 are derived using data on fuel use in road transport (from https://www.iea.org) supported by national data on vehicle numbers and assumptions of per-vehicle mileage travelled. Considering explicitly vehicle-type-specific emission rates and respective activity data allows for better reflection of often significant regional differences in fleet structure, that is, large number of motorcycles in South and South-East Asia and generally lower car ownership numbers in parts of the developing world. GAINS emissions are distributed globally (0.5° × 0.5°) using road network data, assumptions about road-type vehicle density and population data.

The vehicle-type-specific TWP and BWP emission factors draw on review of several measurement papers (Klimont et al.^77^) that were recently updated^52^ using primarily Denier van der Gon et al.^78^, EEA^79^ and Harrison et al.^41^. There are large uncertainties in emission factors including the PM size distribution. GAINS assumes that PM10 TWPs represent ~10% and PM2.5 ~1% of total TWPs, whereas PM10 BWPs is ~80% and PM2.5 is 40–50% of total BWPs independently on vehicle type^77^. Here, we assumed that 2.5, 5, 10, 20 and 40% of the total TWPs and 60, 70, 80, 90 and 100% of the total BWPs is PM10 (Supplementary Table 1) and then calculated the geometric mean and geometric standard deviation. Accordingly, 0.25, 0.5, 1, 2 and 4% of total TWPs and 30, 40, 50, 60 and 70% of total BWPs were assumed PM2.5 (Supplementary Table 1), based on the range of values reported in the literature (Table 3.96 and 3.97 in Klimont et al.^77^ and references therein).

### Atmospheric transport modelling of road microplastics

The gridded TWP emissions were adopted to the Lagrangian particle transport model FLEXPART (FLEXible PARTicle Dispersion Model) version 10.4^80–83^. The model was set to run in forward mode for year 2014 with a spin-up period of 1 month (December 2013). Boundary layer turbulent mixing and convection processes affecting particle transport in clouds are parameterized in the model^80,84^. The model was driven by 3-hourly 1° × 1° operational analyses from the European Centre for Medium-Range Weather Forecast, the spatial output resolution of concentration and deposition fields was set to 0.5° × 0.5° in a global domain with a daily temporal resolution.

FLEXPART assumes a spherical shape of particles^80^ and, as such, the dispersion of road microplastics was modelled. One of the most uncertain aspects of the TWP and BWP emissions is their size distribution. It depends on different properties of the tyre, driving operation and composition and texture of the pavement^21^. Mathissen et al.^85^, Sanders et al.^74^ and Kumar et al.^73^ reported that TWP and BWP can be even emitted as ultrafine particles due to thermomechanical processes. A bimodal size distribution for TWP has been suggested with one maximum in the fine mode and another in the coarse mode^86–89^. On the contrary, an unimodal size distribution has been reported for BWP with maxima ranging between 1.0 and 6.0 μm^23,30,41,74,90^ (Supplementary Fig. 4).

Model simulations were carried out for each of the above emission scenarios (five). However, since also the size distribution within the PM10 and PM2.5 modes is uncertain, we simulated particle transport for three different particle sizes in the PM2.5 (0.5, 1.0 and 2.1 μm) and five in the PM10 mode (0.5, 2.1, 3.2, 6.0 and 9.5 μm) and applied a range of different a posteriori weightings of these size classes. The respective eight mass fractions for TWPs and BWPs used in FLEXPART are shown in Supplementary Fig. 4.

Yet another source of uncertainty is the efficiency with which particles are scavenged by precipitation. Plastics are generally hydrophobic and should therefore be rather inefficient cloud condensation nuclei (CCN)^91,92^. However, as known for BC, coatings may make the particles more hydrophilic with time in the atmosphere^49^. The efficiency of aerosols to serve as ice nuclei (IN) is also not well known. To bracket this type of uncertainty in our simulations, we accounted for three different in-cloud scavenging properties (low, medium and high CCN/IN efficiency, Supplementary Table 2) in each of the aforementioned particle sizes. We report simulated concentrations and deposition amounts as the geometric mean values of the 120 ensemble members (five assumptions of the airborne fraction, eight for the size distribution, three for the CCN/IN efficiency) and quantify their uncertainty as their geometric standard deviation.

The simulations also accounted for below-cloud scavenging and dry deposition, assuming a particle density for TWPs of 1234 kg m^−3^, which is in the middle of the densities of 945 kg m^−3^ for natural rubber and 1522 kg m^−3^ for synthetic rubber^93,94^. This density is within the reported range for microplastics (940–2400 kg m^−3^)^95^. For BWPs a higher density was assumed (2000 kg m^−3^) considering that BWP may also contain metals^22^. These values were held constant for all ensemble members.

### Atmospheric lifetimes of road microplastics

The modelled lifetime (*τ*) of of road microplastic particles in each grid cell is identical to the lifetime due to transport (*t*_trans_) in and out of the aforementioned grid cell, chemical loss (*t*_chem_) and deposition (*t*_depo_)

$$\frac{1}{\tau } = \frac{1}{{{t}_{{\mathrm{trans}}}}} + \frac{1}{{{t}_{{\mathrm{chem}}}}} + \frac{1}{{{t}_{{\mathrm{depo}}}}}.$$

For road microplastics, we assume no chemical interactions of TWPs and BWPs. The modelled lifetime in FLEXPART can be written by the species mass balance equation of Croft et al.^96^ as follows:

$$\frac{{{\mathrm{d}}{\mathbf{C}}(t)}}{{{\mathrm{d}}t}} = {\mathbf{S}}\left( t \right) - \frac{{{\mathbf{C}}(t)}}{{\tau (t)}},$$

where **C**(*t*) is the atmospheric burden of road microplastics at time *t*, **S**(*t*) is the time-dependent source emission fluxes and *τ*(*t*) is the removal timescale. Assuming steady-state conditions and considering that emission fluxes are continuous, there is a quasi-equilibrium between sources and removals; hence, the modelled lifetime *τ*_mod_ can be defined as:

$$\tau _{\mathrm{mod}} = {\mathbf{C}}_{\mathrm{mp}}/{\mathbf{L}}_{\mathrm{mp}}^{{\mathrm{trans}},{\mathrm{chem}},{\mathrm{depo}}},$$

where **C**_mp_ is the atmospheric burden of road microplastics and ${\mathbf{L}}_{\mathrm{mp}}^{{\mathrm{trans}},{\mathrm{chem}},{\mathrm{depo}}}$ is the total loss due to any process affecting TWPs and BWPs in the model (transport, chemistry, deposition).

### Statistics and uncertainty calculations

We plot the probability density functions (PDFs) for deposition of TWPs and BWPs that resulted from all the ensemble members of our sensitivity in Supplementary Fig. 5. In the present case, five ensemble members represented the uncertainty in the emissions (Supplementary Table 1), eight that are in the size distribution (Supplementary Fig. 4) and three members that are in the CCN/IN efficiency (Supplementary Table 2), which gives a total of 120 ensemble members for each size (PM2.5 and PM10). Supplementary Figure 5 shows that deposition follows a log-normal distribution with a PDF that can be expressed as follows:

$$f\left( {\chi ,\mu _{\mathrm{g}},\sigma _{\mathrm{g}}} \right) = \frac{1}{{\chi \sigma _{\mathrm{g}}\sqrt {2\pi } }}{\mathrm{exp}}\left( { - \frac{{(\ln\,x - \mu _{\mathrm{g}})^2}}{{2\sigma _{\mathrm{g}}^2}}} \right),$$

where *χ* is the random variable, and *μ*_g_ and *σ*_g_ are the mean and standard deviation of the distribution of ln *χ*. This relationship is true regardless of the base of the logarithmic or exponential function^97^. Thus, the results can be expressed by the geometric mean (*μ*_g_) and the uncertainty by the geometric standard deviation (*σ*_g_) of *χ*, which are given below:

$$\mu _{\mathrm{g}} = \root {N} \of {{{\mathbf{A}}_1{\mathbf{A}}_2 \ldots {\mathbf{A}}_{\mathbf{N}}}}\;{\mathrm{and}}\;\sigma _{\mathrm{g}} = {\mathrm{exp}}\left( {\sqrt {\frac{{\mathop {\sum }\nolimits_{i = 1}^N \left( {\ln \frac{{{\mathbf{A}}_{\mathbf{i}}}}{{\mu _{\mathrm{g}}}}} \right)^2}}{N}} } \right),$$

where ${\mathbf{A}}_1,{\mathbf{A}}_2, \ldots ,{\mathbf{A}}_{\mathbf{N}}$ are the results from each ensemble member and *N* the size of the ensemble (120 members for the PM2.5 and PM10 mode, respectively, for each of the TWP and BWP).

The geometric standard deviation is a dimensionless multiplicative factor, also called geometric SD factor^98^. We present resulting concentrations and deposition here with geometric SD factor in conjunction with geometric mean as “the range from “the geometric mean divided by the geometric SD factor” to “the geometric mean multiplied by the geometric SD factor”, rather add/subtract “geometric SD factor” to/from “geometric mean”^99^.

## Supplementary information

Supplementary Information

Peer Review File

Supplementary Data

Supplementary Movie 1

Supplementary Movie 2

Supplementary Movie 3

Supplementary Movie 4

Supplementary Movie 5

## Data Availability

All primary sources (TWP and BWP emission data) are publicly available in 10.5061/dryad.qrfj6q5bx (temporary link: https://datadryad.org/stash/share/_dEIxj28-AHDSoIEPIRdufbljARA-NLyKYOs2n2CqqE). FLEXPART version 10.4 model is publicly available^80^. Operational meteorological data that were used in FLEXPART version 10.4 model can be downloaded directly from the European Centre for Medium-Range Weather Forecasts (ECMWF, https://www.ecmwf.int) following their rules and regulations. All FLEXPART version 10.4 simulation results can be found in 10.5061/dryad.qrfj6q5bx or upon request to N.E. The same dataset also contains land–sea, ocean, continental and country masks (Supplementary Fig. 6) that were used in the calculations of continental emissions, oceanic deposition and transport efficiencies, together with the ECMWF data of sea-ice area fraction, snow depth (for the definition of mountains), snowfall and total precipitation that were used in the calculations of snow concentrations.

## Code availability

The Lagrangian particle dispersion model FLEXPART is open access and can be obtained from https://www.flexpart.eu. All figures were created using open access Python 3 programming language. The post-processing codes can be obtained from the corresponding author upon request.
