# Supplementary material for: Atmospheric transport is a major pathway of microplastics to remote regions
Source: Nat Commun. 2020 Jul 14;11:3381. doi: 10.1038/s41467-020-17201-9 (PMC7360784; doi:10.1038/s41467-020-17201-9)
Supplement: Supplementary file 1 — Supplementary Information [file 41467_2020_17201_MOESM1_ESM.pdf]

# Supplementary Information (Nature Communications)

## Atmospheric transport is a major pathway of microplastics to remote regions

**N. Evangeliou *et al.***

**Supplementary notes**

All figures have been created using the open access general-purpose programming language Python version 3.

Snow concentrations were calculated using daily fields of sea-ice area fraction and total snowfall from European Centre for Medium Range Weather Forecast (ECMWF), which are all open access (see Data availability). All continental regions and marine/oceanic regions described in the manuscript are also provided (Supplementary Figure 6, Data availability). Mountain regions were defined using operational snow depth data from ECMWF (see Data availability), only where snow depth was positive throughout the year.

**Supplementary Table 1.** Mass fractions of PM2.5 and PM10 size modes of TWPs and BWPs with respect to total TWPs and BWPs assumed in the emissions ingested in FLEXPART model. Five scenarios were created assuming that 2.5%, 5%, 10%, 20% and 40% of the total TWP were emitted as PM10 and 0.25%, 0.5%, 1%, 2% and 4% as PM2.5. Accordingly, 60%, 70%, 80%, 90% and 100% of the total BWPs were assumed PM10 and 30%, 40%, 50%, 60% and 70% were assumed PM2.5, based on the range of values reported in the literature. These values were used in the ensemble of 120 members (Methods) together with assumptions on particle size distribution (eight for each of the PM2.5 and PM10 fractions, Supplementary Figure 4) and CCN/IN efficiency (three different sets of scavenging coefficients per fraction, Supplementary Table 2).

| TWPs | PM2.5 | PM10 | BWPs | PM2.5 | PM10 |
|---|---|---|---|---|---|
| Scenario 1 | 0.25% | 2.5% | Scenario 1 | 30% | 60% |
| Scenario 2 | 0.5% | 5% | Scenario 2 | 40% | 70% |
| Scenario 3 | 1% | 10% | Scenario 3 | 50% | 80% |
| Scenario 4 | 2% | 20% | Scenario 4 | 60% | 90% |
| Scenario 5 | 4% | 40% | Scenario 5 | 70% | 100% |

*Note that PM2.5 is always part of PM10, and PM10 must by definition always be more than PM2.5.

**Supplementary Table 2.** Different scavenging parameters of below–cloud and in–cloud scavenging used in FLEXPART version 10.4 for the ensemble model simulations of microplastics. $A$ and $B$ are rain and snow collection efficiencies for below-cloud scavenging, $A_i$ is the cloud condensation nuclei (CCN) efficiency and $B_i$ the ice nuclei (IN) efficiency that are used in in-cloud scavenging following Grythe et al.[1]. These values were used in the ensemble of 120 members (Methods) together with different assumption for the airborne fraction (five for each of the PM2.5 and PM10 fractions, Supplementary Table 1) and particle size distribution (eight for each of the PM2.5 and PM10 fractions, Supplementary Figure 4).

| | $\boldsymbol{A}$ | $\boldsymbol{B}$ | $\boldsymbol{A_i}$ | $\boldsymbol{B_i}$ |
|---|---|---|---|---|
| Low efficiency | 1 | 1 | 0.001 | 0.01 |
| Medium efficiency | 1 | 1 | 0.05 | 0.15 |
| High efficiency | 1 | 1 | 0.5 | 0.8 |

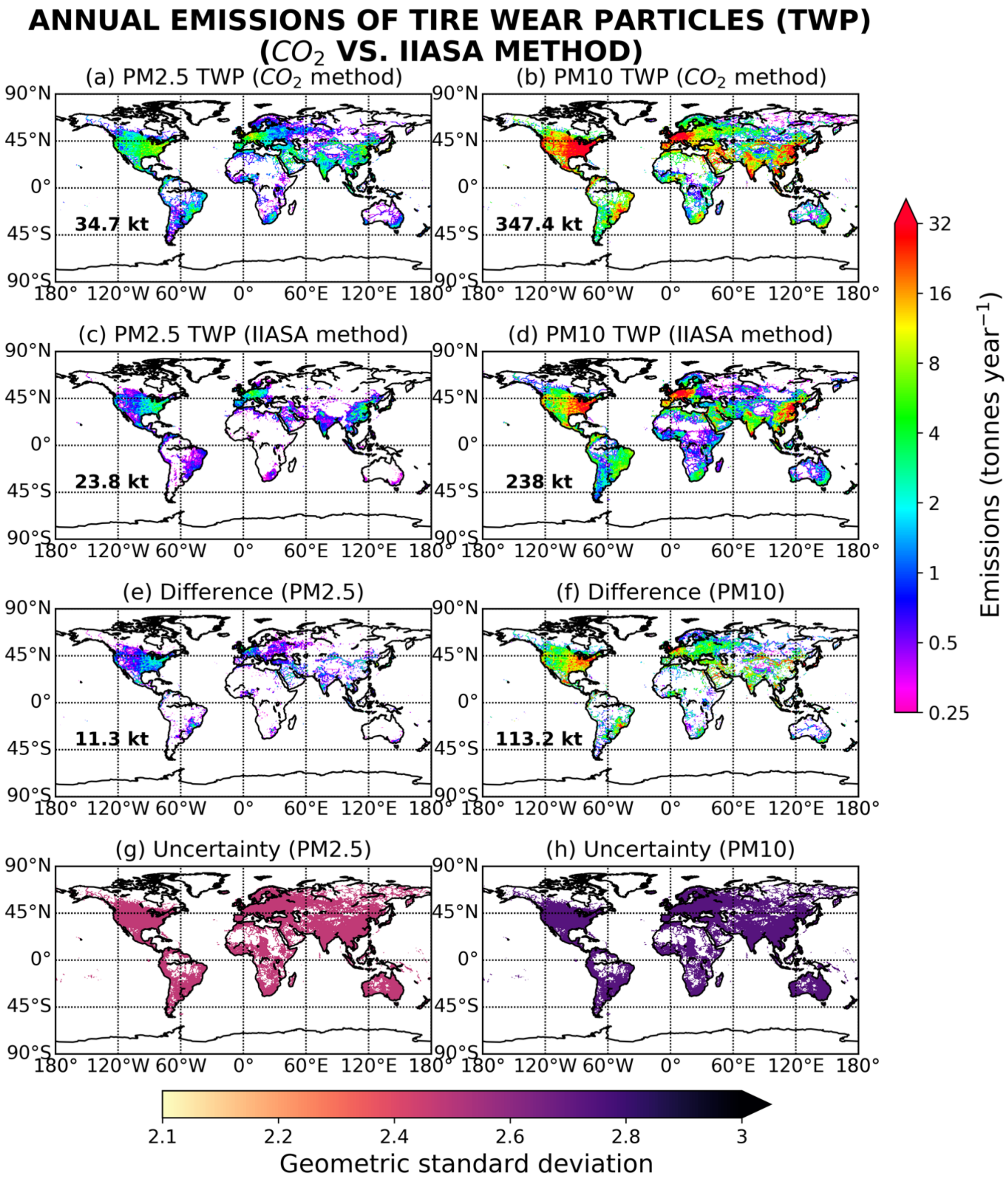


**Supplementary Figure 1.** Annual emissions of TWPs with the CO2 ratio method (panels a and b) and using the GAINS model (panels c and d) for PM2.5 and PM10 particles, respectively (Fig. 1 and Methods). Emissions were calculated as the geometric mean of the five scenarios for the airborne fraction of total TWPs, assuming 2.5%, 5%, 10%, 20% and 40% of the total TWP are emitted as PM10 and 0.25%, 0.5%, 1%, 2% and 4% as PM2.5 following a log-normal distribution (Supplementary Table 1). Difference in emissions using the two different methodologies are presented in panels e and f. Uncertainties for the PM2.5 and PM10 TWP emissions (panels g and h) were calculated as the geometric standard deviation of the five assumed different airborne fractions per size mode (PM2.5 and PM10) with respect to total TWP emissions (Methods). Bold numbers at the lower left side of panels a–d represent total annual emissions of TWPs, whereas bold numbers at the lower left side of panels e and f are the respective annual differences in the emissions of TWPs from the two methodologies used.

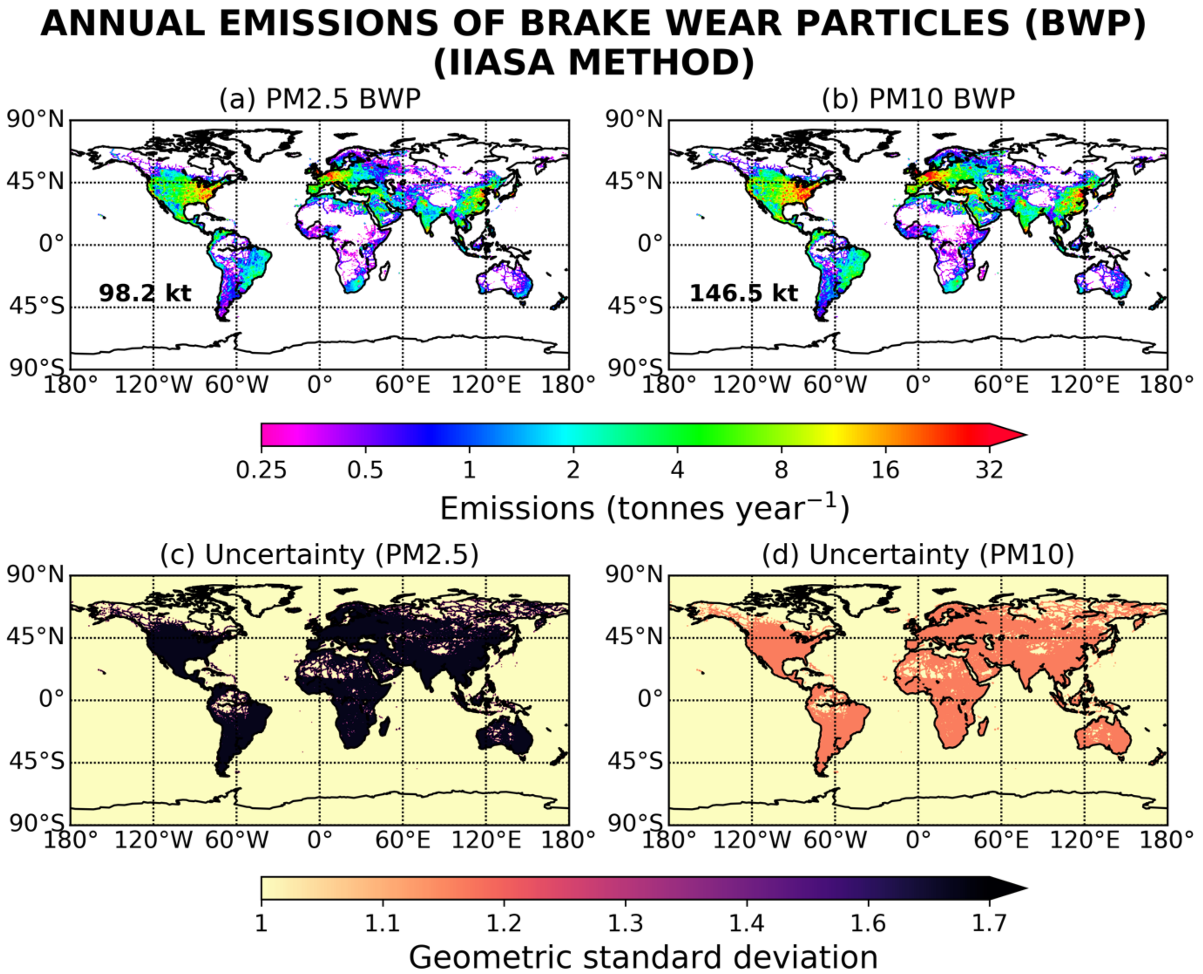


**Supplementary Figure 2.** Annual emissions of BWPs from the GAINS model (panels a and b) for PM2.5 and PM10, respectively. The emissions are the geometric mean of five different assumptions on the airborne fraction for each size bin (30%, 40%, 50%, 60% and 70% of total BWPs were assumed to be PM2.5 and 60%, 70%, 80%, 90% and 100% of the total BWPs to be PM10) following a log-normal distribution (Supplementary Table 1). The estimated associated uncertainty (panels c and d) is expressed with the geometric standard deviation of the aforementioned scenarios (Methods). Bold numbers at the lower left side of panels a and b represent total annual emissions of BWPs.

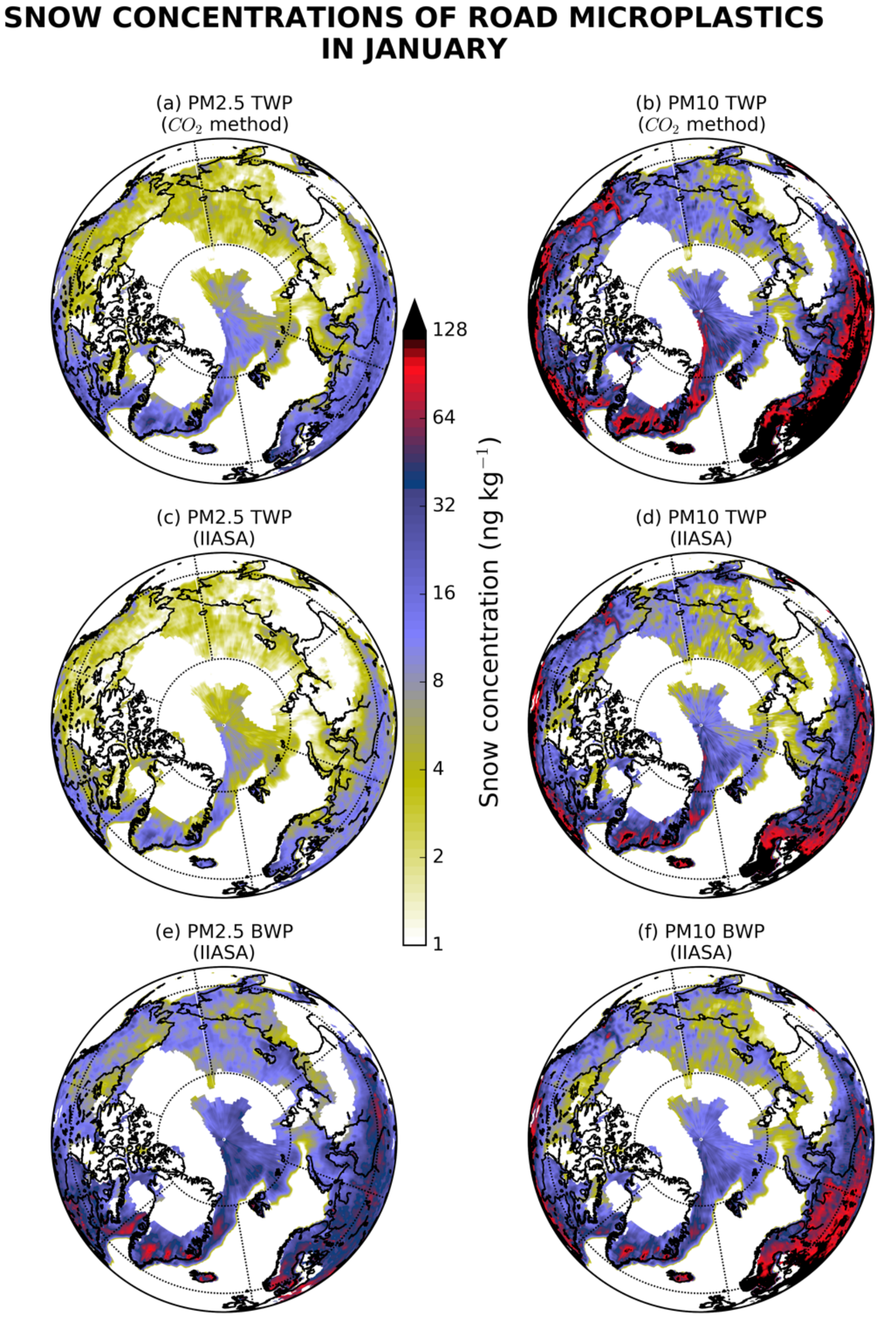


**Supplementary Figure 3.** Monthly (12) snow concentrations of road microplastic particles in the Arctic snow. Total snowfall and snow depth were adopted from European Centre for Medium Range Weather Forecast (ECMWF). The concentrations were calculated using daily modelled deposition of road microplastics and daily fields of total snowfall (in m of water equivalent) from ECMWF operational fields and only over land (using a land-sea mask) and/or sea-ice (using sea-ice area fraction from ECMWF). The annual average snow concentrations were calculated only for months where snowfall was more than 90% of total precipitation (Methods).

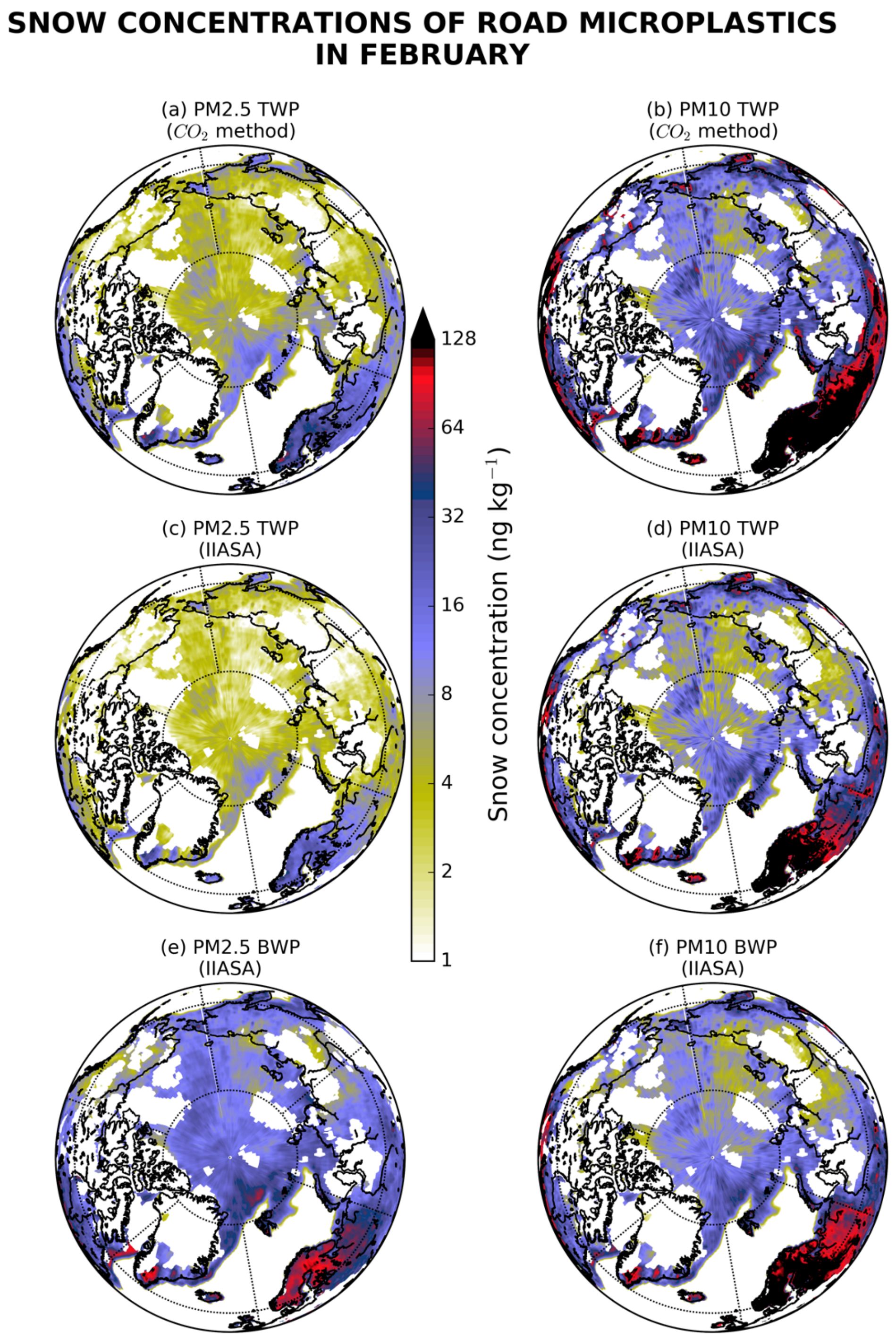


**Supplementary Figure 3.** *Continued.*

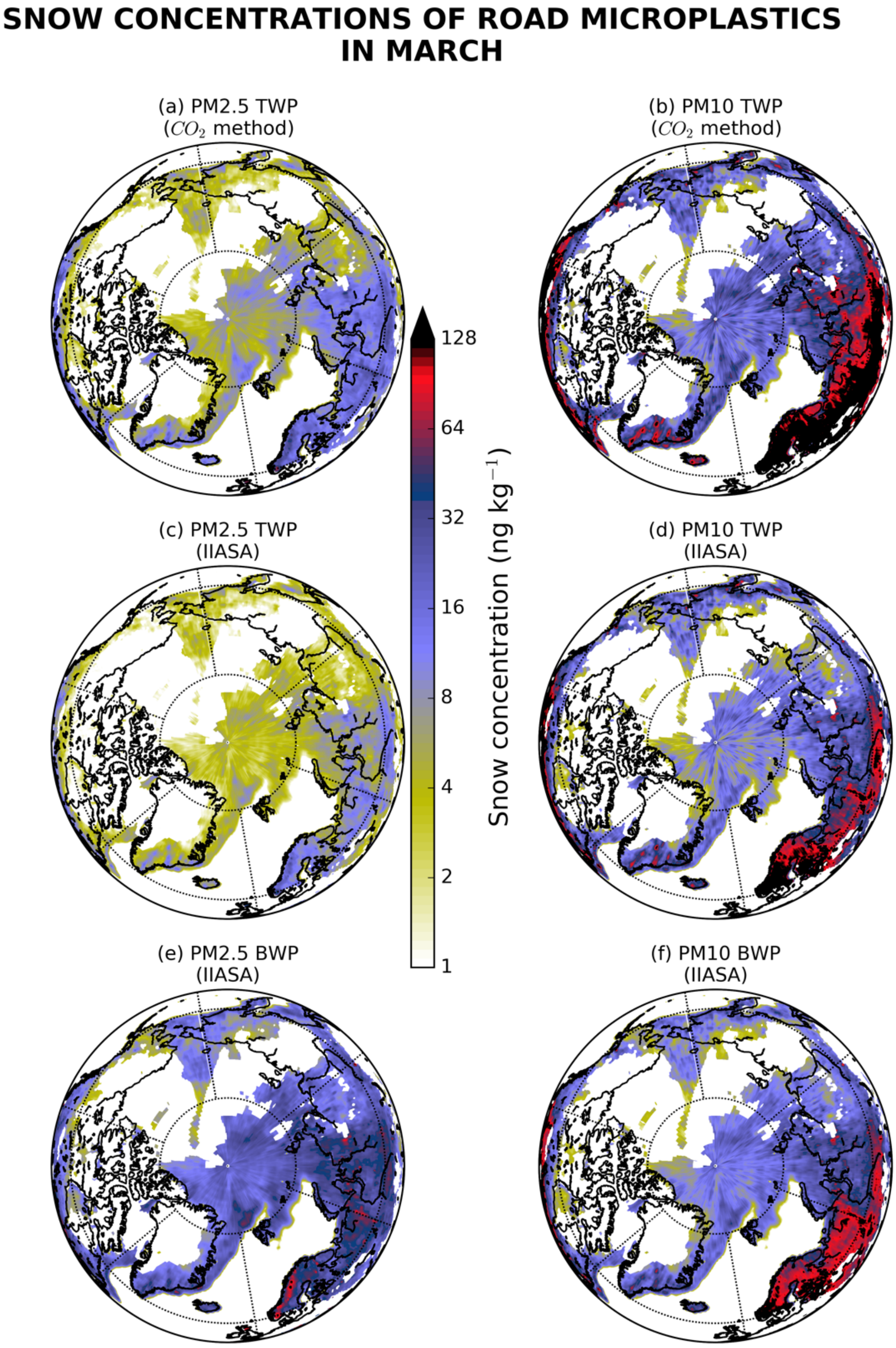


**Supplementary Figure 3.** *Continued.*

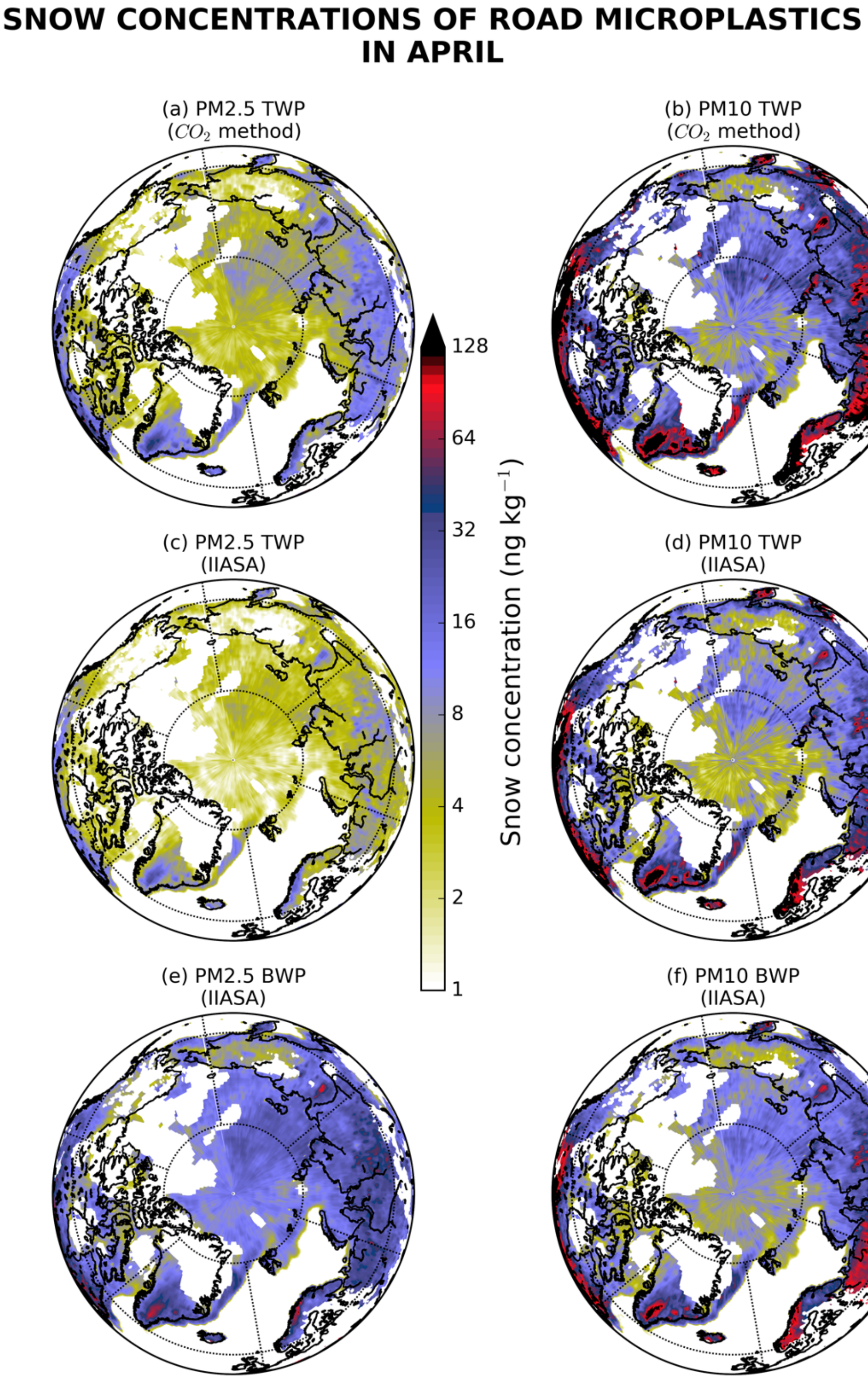


**Supplementary Figure 3.** *Continued.*

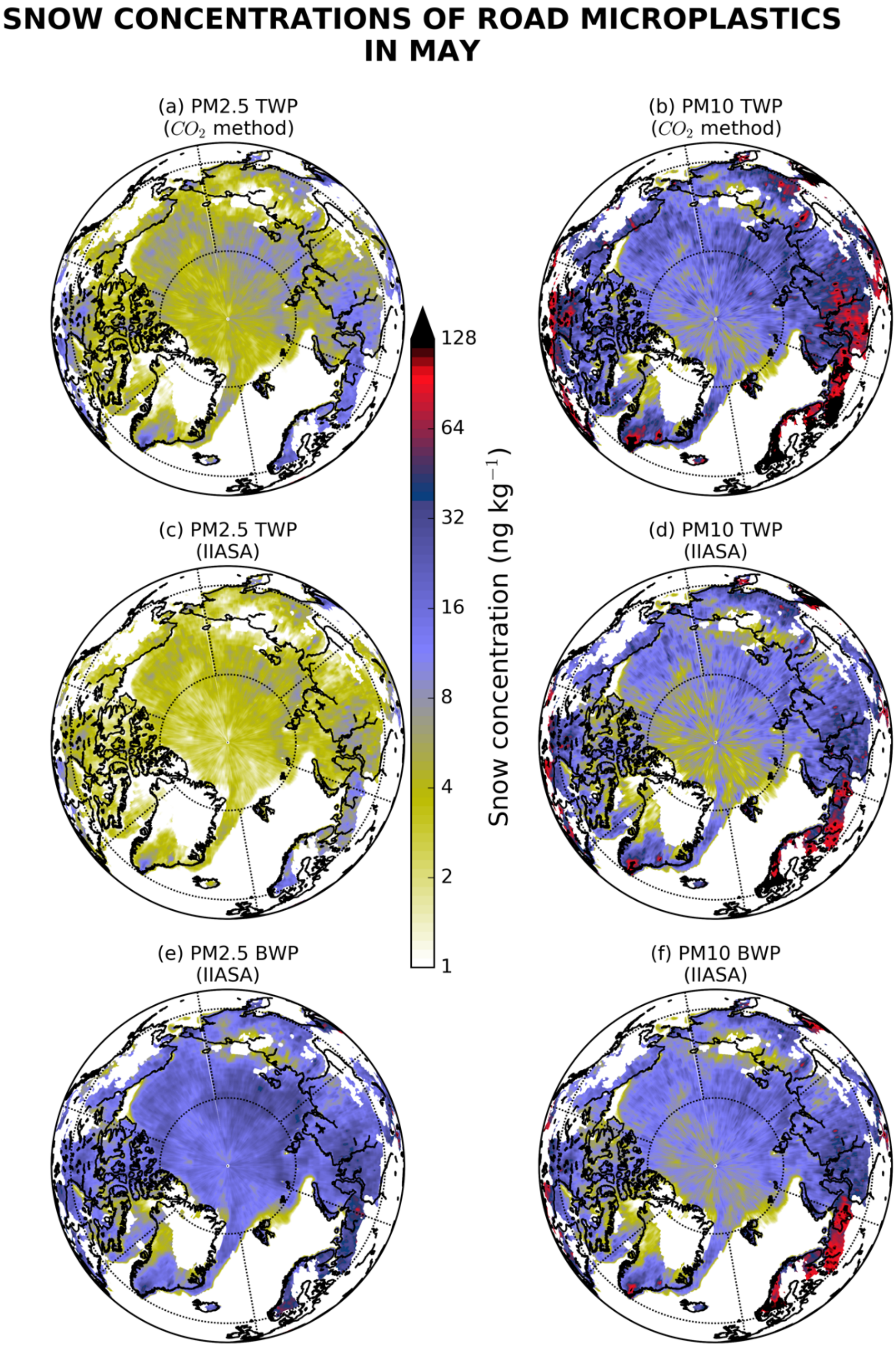


**Supplementary Figure 3.** *Continued.*

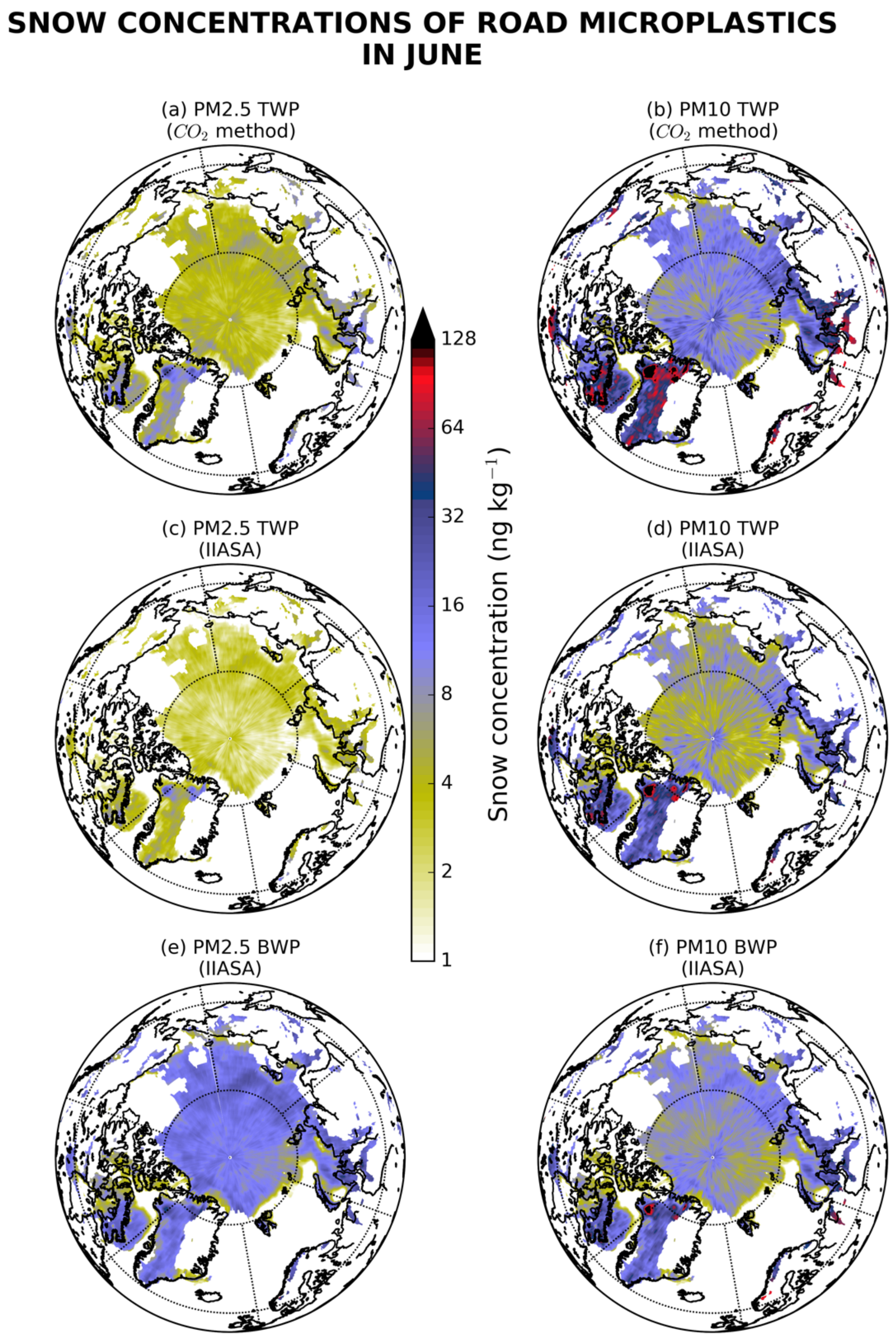


**Supplementary Figure 3.** *Continued.*

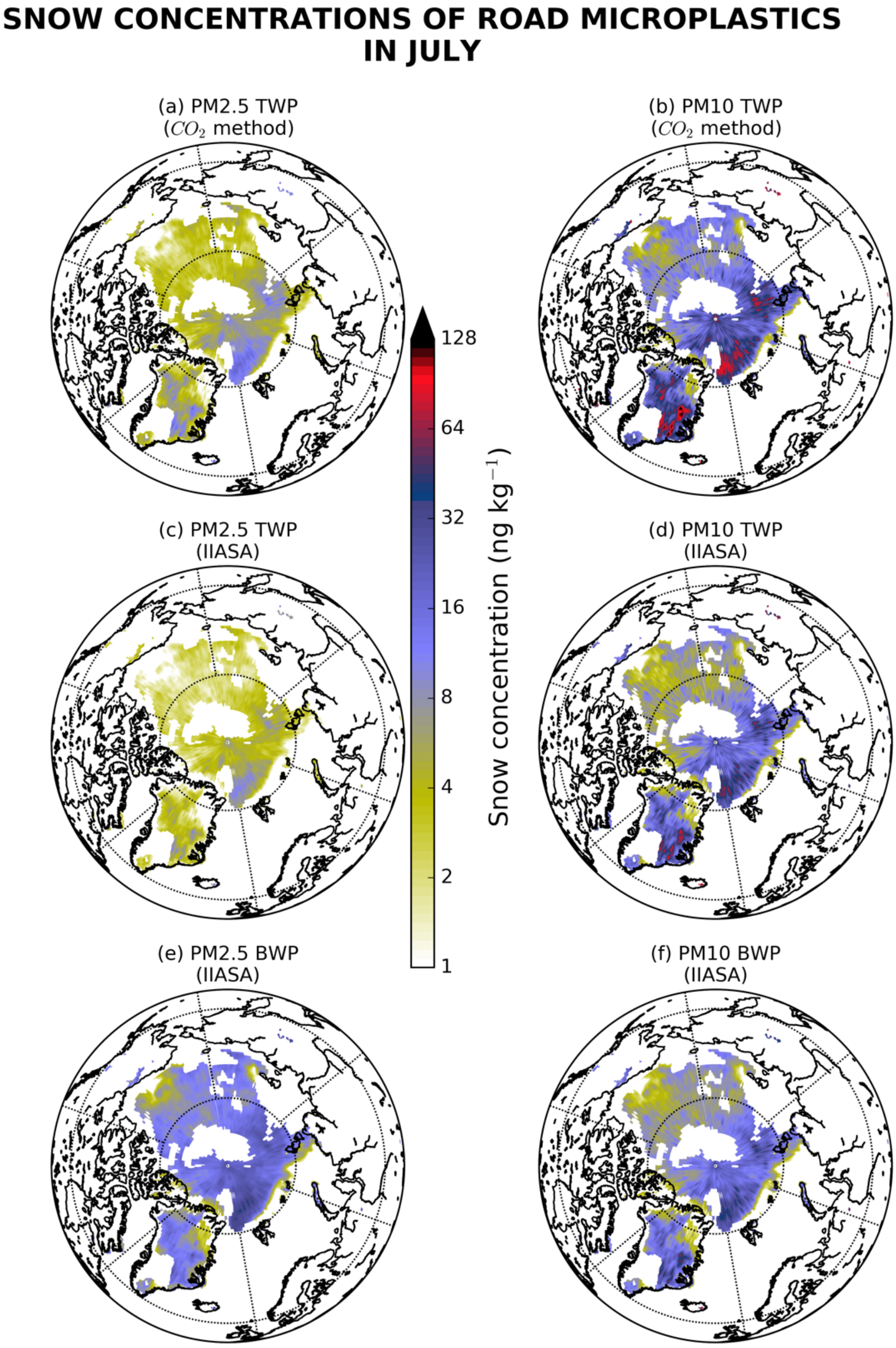


**Supplementary Figure 3.** *Continued.*

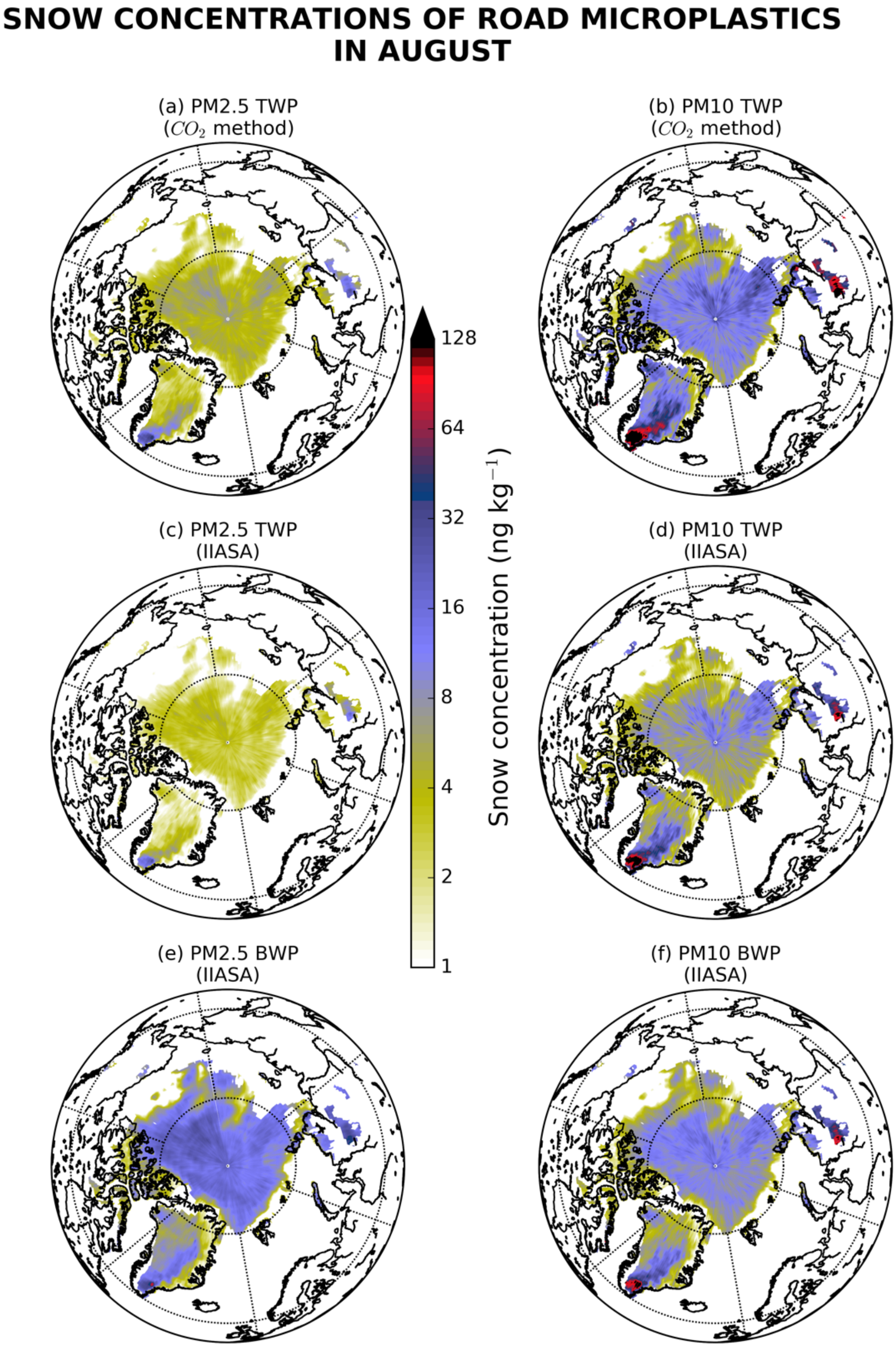


**Supplementary Figure 3.** *Continued.*

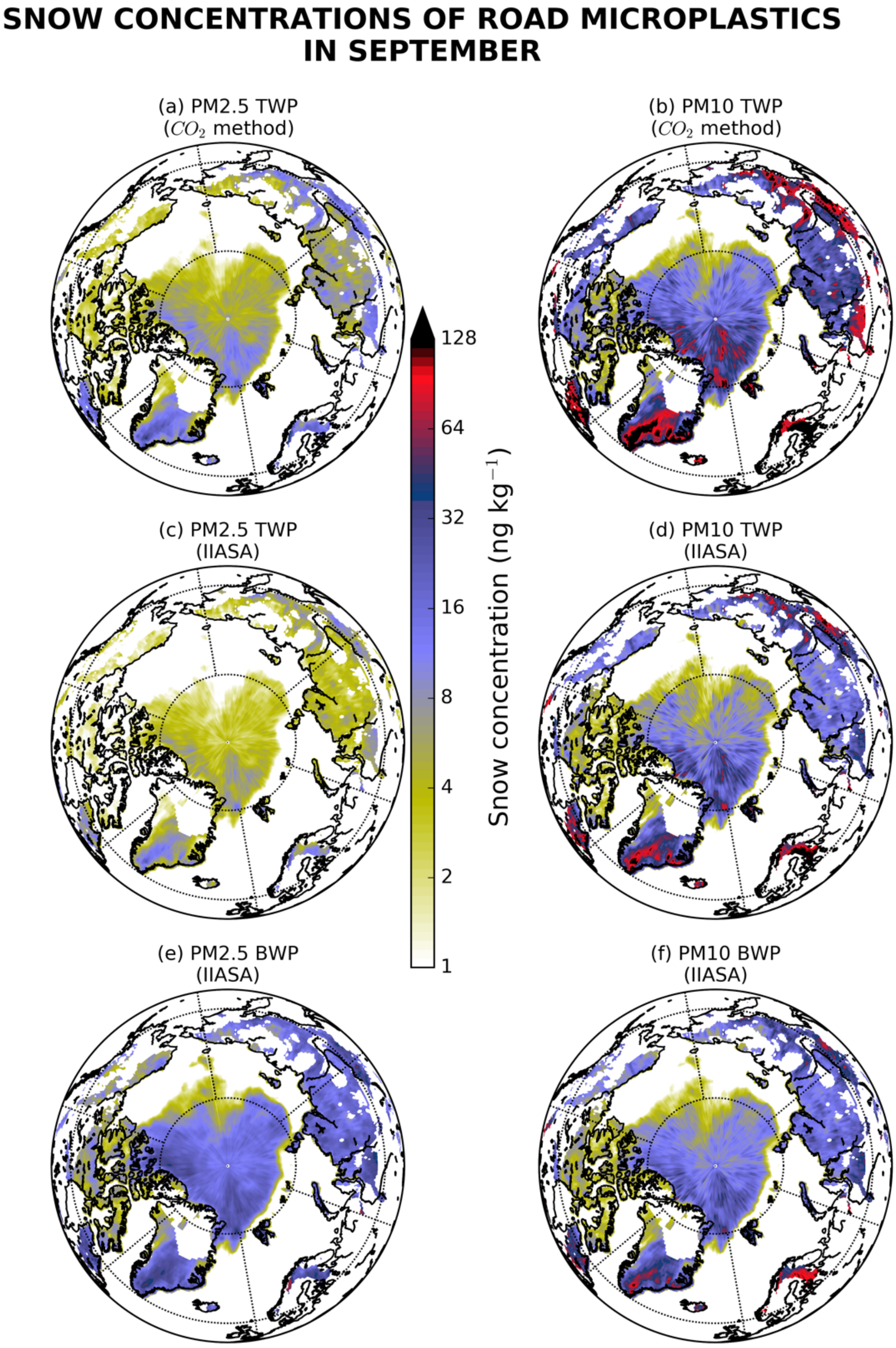


**Supplementary Figure 3.** *Continued.*

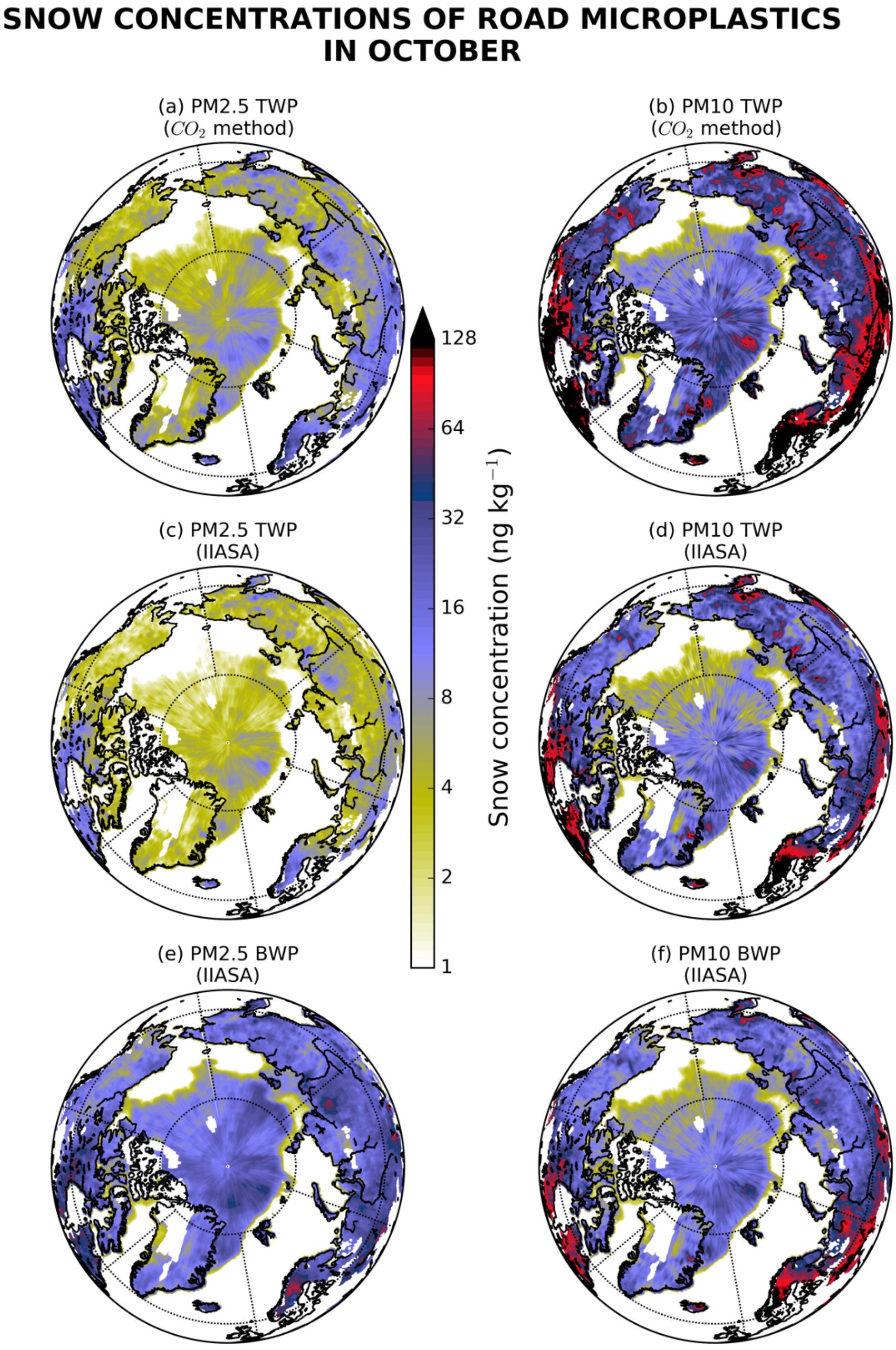


**Supplementary Figure 3.** *Continued.*

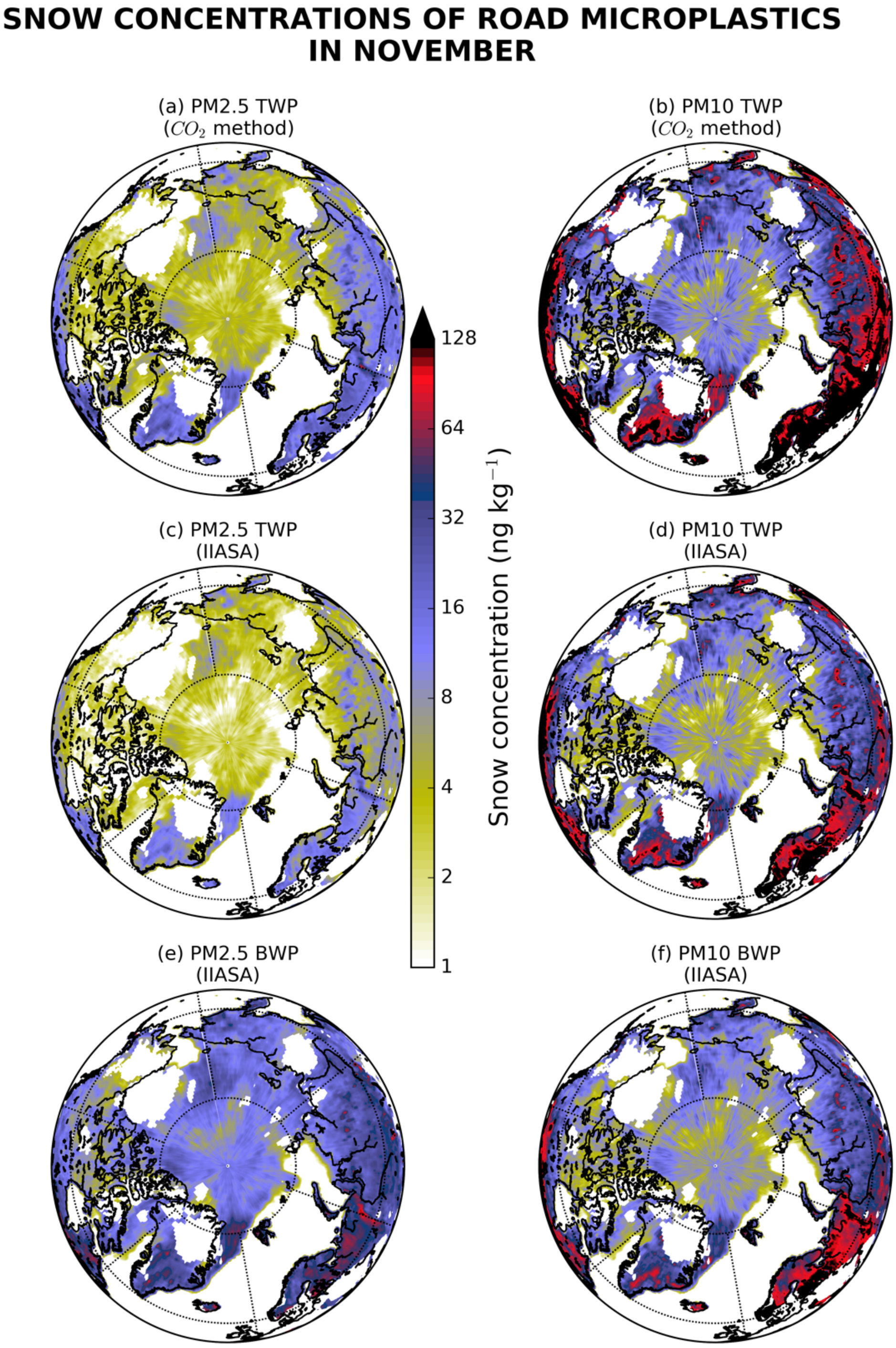


**Supplementary Figure 3.** *Continued.*

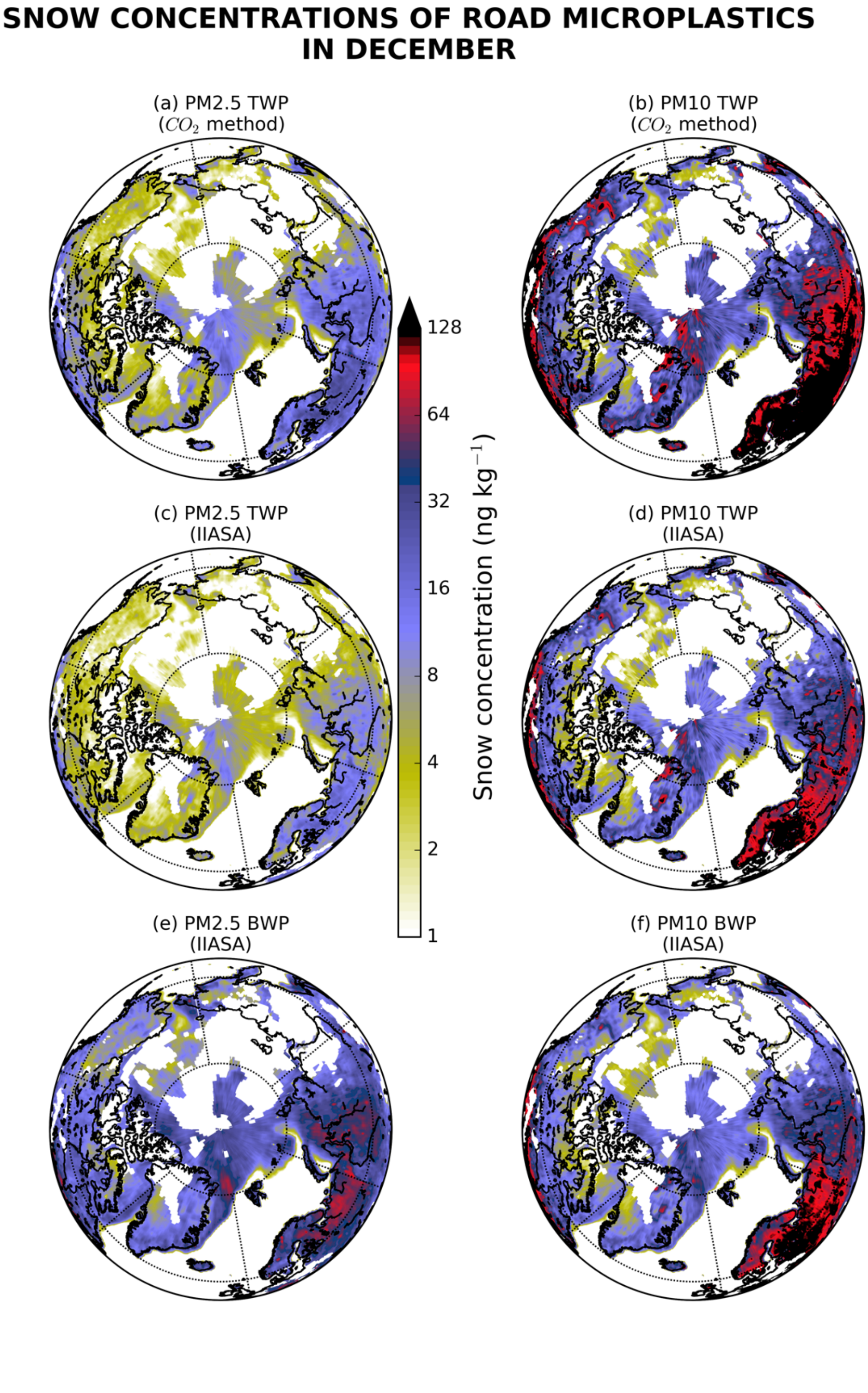


**Supplementary Figure 3.** *Continued.*

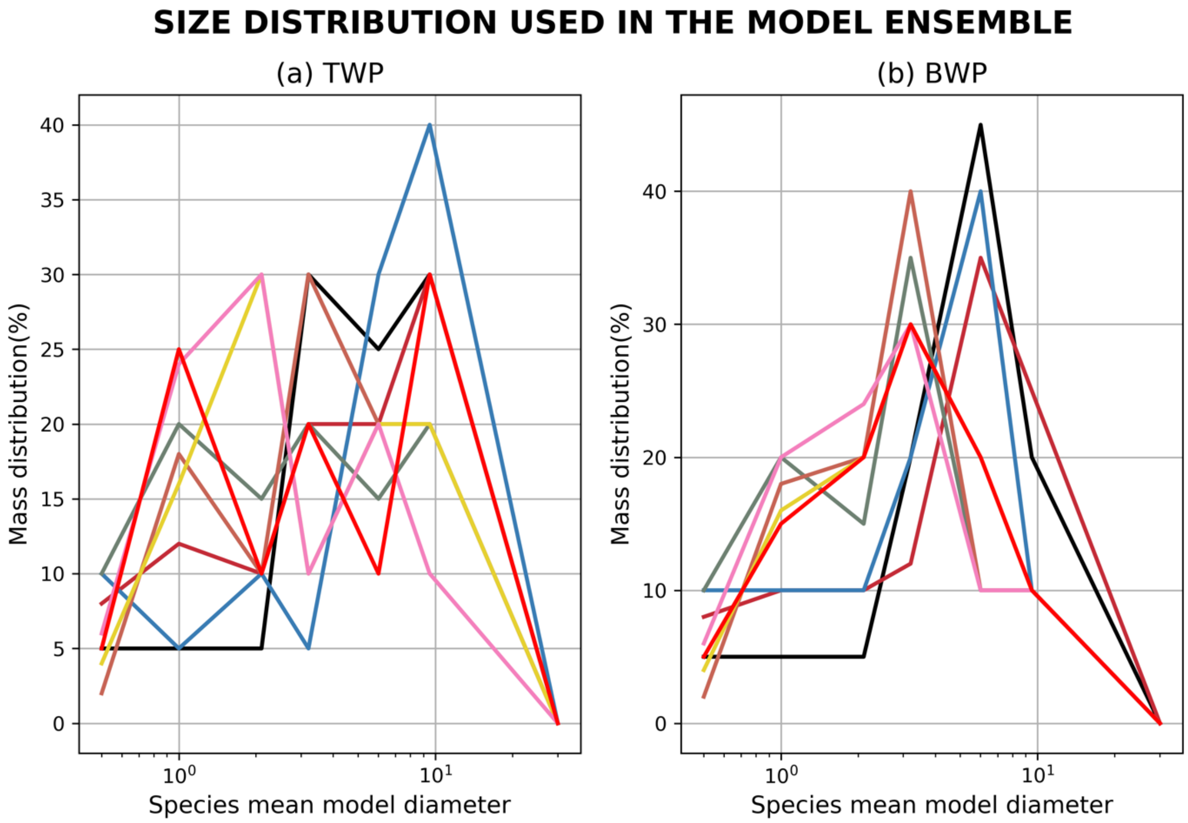


**Supplementary Figure 4.** Size distribution used in the simulations of road microplastics (TWPs and BWPs) presenting a set of different a posteriori weightings (mass fractions) for different size classes (eight for each of the TWP and BWP simulations). Three size classes were used for PM2.5 (0.5, 1.0 and 2.1 μm) and five for the PM10 mode (0.5, 2.1, 3.2, 6.0 and 9.5 μm). Note the bimodal (two peaks)[2–5] size distribution of TWPs with one maximum close to the fine mode and another in the coarse mode and the unimodal (single peak)[6–10] distribution of BWPs with maximum in the fine or coarse mode.

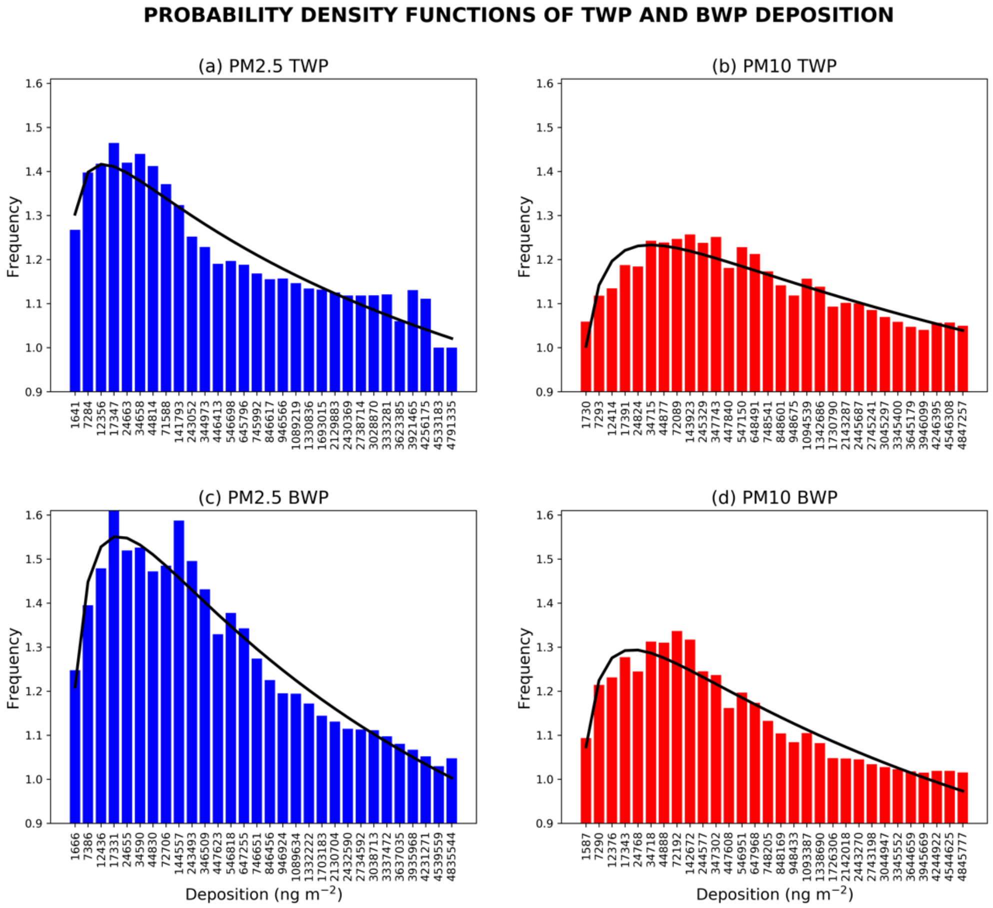


**Supplementary Figure 5.** Probability density functions (PDF) of deposition of TWPs and BWPs for both PM2.5 and PM10 sizes. Note that PDF is based on a model ensemble of 120 members that includes five (5) members with different assumptions on the airborne fraction in the emissions (Supplementary Table 1), eight (8) members assuming different particle size distribution in the atmospheric dispersion (Supplementary Figure 4) and three (3) members with different scavenging coefficients expressing the CCN/IN efficiency (Supplementary Table 2).

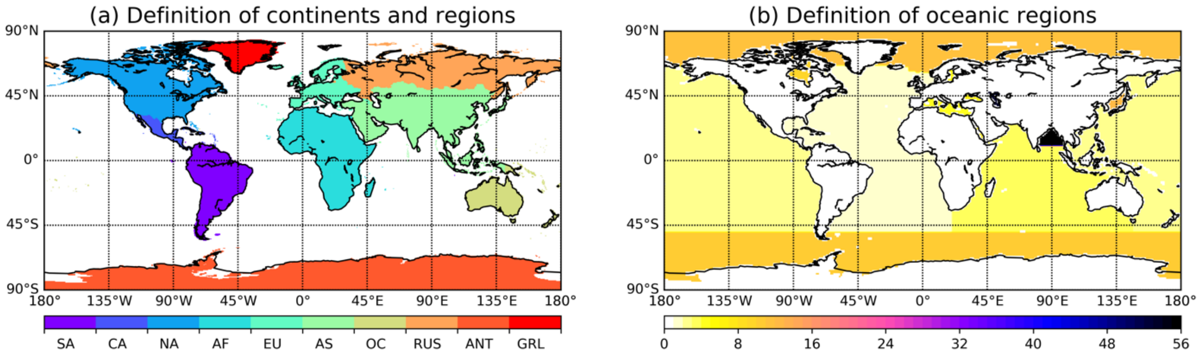


**Supplementary Figure 6.** (a) Masked continents used in the present study for South America (SA), Central America (CA), North America (NA), Africa (AF), Europe (EU), Asia (AS), Oceania (OC), Russia (RUS), Antarctica (ANT) and Greenland (GRL). (b) Masks of oceanic regions used in the present study. Values between 1 and 56 include Atlantic Ocean, Pacific Ocean, Indian Ocean, Mediterranean Sea, Baltic Sea, Black Sea, Red Sea, Persian Gulf, Hudson Bay, Southern Ocean, Arctic Ocean, Sea of Japan, Kara Sea, Sulu Sea, Baffin Bay, East Mediterranean, West Mediterranean, Sea of Okhotsk, Banda Sea, Caribbean Sea, Andaman Basin, North Caribbean, Gulf of Mexico, Beaufort Sea, South China Sea, Barents Sea, Celebes Sea, Aleutian Basin, Fiji Basin, North American Basin, West European Basin, Southeast Indian Basin, Coral Sea, East Indian Basin, Central Indian Basin, Southwest Atlantic Basin, Southeast Atlantic Basin, Southeast Pacific Basin, Guatemala Basin, East Caroline Basin, Marianas Basin, Philippine Sea, Arabian Sea, Chile Basin, Somali Basin, Mascarene Basin, Crozet Basin, Guinea Basin, Brazil Basin, Argentine Basin, Tasman Sea, Atlantic Indian Basin, Caspian Sea, Sulu Sea II, Venezuela Basin, Bay of Bengal, Java Sea, East Indian Atlantic Basin. Note that most of the smaller regions have been overwritten and are not visible.

**Supplementary references**


1. Grythe, H. *et al.* A new aerosol wet removal scheme for the Lagrangian particle model FLEXPARTv10. *Geosci. Model Dev.* **10**, 1447–1466 (2017).

2. Fukahori, Y. & Yamazaki, H. Mechanism of rubber abrasion. Part I: Abrasion pattern formation in natural rubber vulcanizate. *Wear* **171**, 195–202 (1994).

3. Gustafsson, M. *et al.* Properties and toxicological effects of particles from the interaction between tyres, road pavement and winter traction material. *Sci. Total Environ.* **393**, 226–240 (2008).

4. Wang, Q., Zhang, Q., Wu, Y. & Wang, X. C. Physicochemical conditions and properties of particles in urban runoff and rivers: Implications for runoff pollution. *Chemosphere* **173**, 318–325 (2017).

5. Kreider, M. L., Panko, J. M., McAtee, B. L., Sweet, L. I. & Finley, B. L. Physical and chemical characterization of tire-related particles: Comparison of particles generated using different methodologies. *Sci. Total Environ.* **408**, 652–659 (2010).

6. Sanders, P. G., Xu, N., Dalka, T. M. & Maricq, M. M. Airborne brake wear debris: Size distributions, composition, and a comparison of dynamometer and vehicle tests. *Environ. Sci. Technol.* **37**, 4060–4069 (2003).

7. Iijima, A. *et al.* Particle size and composition distribution analysis of automotive brake abrasion dusts for the evaluation of antimony sources of airborne particulate matter. *Atmos. Environ.* **41**, 4908–4919 (2007).

8. Mosleh, M., Blau, P. J. & Dumitrescu, D. Characteristics and morphology of wear particles from laboratory testing of disk brake materials. *Wear* **256**, 1128–1134 (2004).

9. Kukutschová, J. *et al.* On airborne nano/micro-sized wear particles released from low-metallic automotive brakes. *Environ. Pollut.* **159**, 998–1006 (2011).

10. Harrison, R. M., Jones, A. M., Gietl, J., Yin, J. & Green, D. C. Estimation of the contributions of brake dust, tire wear, and resuspension to nonexhaust traffic particles derived from atmospheric measurements. *Environ. Sci. Technol.* **46**, 6523–6529 (2012).
