# Supplementary material for: Atmospheric transport is a major pathway of microplastics to remote regions
Source: Nat Commun. 2020 Jul 14;11:3381. doi: 10.1038/s41467-020-17201-9 (PMC7360784; doi:10.1038/s41467-020-17201-9)
Supplement: Supplementary file 3 — Supplementary Data [file 41467_2020_17201_MOESM3_ESM.pdf]

Description of Additional Supplementary Files (Nature Communications)

Atmospheric transport is a major pathway of microplastics to remote regions

N. Evangeliou *et al.*

File Name: **Supplementary_Movie_1.mp4**
Description: Surface concentrations of TWPs (using emissions calculated with the CO2 ratio method and the GAINS model) and BWPs (GAINS model) in the PM2.5 and PM10 size modes (Methods). Each panel is the geometric mean of 120 different simulations with different airborne fraction assumed (five members for each of the PM2.5 and PM10 fractions, Supplementary Table 1), different particle size distribution (eight members for each of the PM2.5 and PM10 fractions, Supplementary Figure 4) and CCN/IN efficiency (three different sets of scavenging coefficients per fraction, Supplementary Table 2).

File Name: **Supplementary_Movie_2.mp4**
Description: Monthly ratios of snowfall to total precipitation from ECMWF operational fields. We calculated snow concentrations for the grid-cells with non-zero snowfall and only for the months where snowfall was more than 90% of total precipitation. Snow concentrations are the geometric mean values of 120 model simulations that accounted for different airborne fraction (five members for each of the PM2.5 and PM10 fractions, Supplementary Table 1), particle size distribution (eight members for each of the PM2.5 and PM10 fractions, Supplementary Figure 4) and CCN/IN efficiency (three different sets of scavenging coefficients per fraction, Supplementary Table 2) following a log-normal distribution (Methods and Supplementary Figure 5).

File Name: **Supplementary_Movie_3.mp4**
Description:Global column integrated concentrations and accumulated deposition of TWPs in the PM2.5 and PM10 modes. Emissions were calculated using the CO2 ratio method (Methods). Each panel is the geometric mean of 120 different simulations with different airborne fraction assumed (five members for each of the PM2.5 and PM10 fractions), different particle size distribution (eight members for each of the PM2.5 and PM10 fractions) and CCN/IN efficiency (three different sets of scavenging coefficients per fraction) presented in detail in Supplementary Table 1, Supplementary Figure 4 and Supplementary Table 2.

File Name: **Supplementary_Movie_4.mp4**
Description: Same as Supplementary Movie 3, but using emissions from the GAINS model (IIASA) (Methods).

File Name: **Supplementary_Movie_5.mp4**
Description: Global column integrated concentrations and accumulated deposition of BWPs in the PM2.5 and PM10 mode using emissions from the GAINS model. Each panel is the

geometric mean of 120 different simulations with different airborne fraction assumed (five members for each of the PM2.5 and PM10 fractions), different particle size distribution (eight members for each of the PM2.5 and PM10 fractions) and CCN/IN efficiency (three different sets of scavenging coefficients per fraction). All members are presented in detail in Supplementary Table 1, Supplementary Figure 4 and Supplementary Table 2.
