# Supplementary material for: Atmospheric transport is a major pathway of microplastics to remote regions
Source: Nat Commun. 2020 Jul 14;11:3381. doi: 10.1038/s41467-020-17201-9 (PMC7360784; doi:10.1038/s41467-020-17201-9)
Supplement: Supplementary file 2 — Peer Review File [file 41467_2020_17201_MOESM2_ESM.docx]

Peer Review Information

**Journal:** Nature Communications

**Manuscript title:** Atmospheric transport, a major pathway of microplastics to remote regions

**Corresponding author name(s):** Dr Nikolaos Evangeliou

**Editorial notes:**

**Transferred manuscripts**

This manuscript has been previously reviewed at another journal that is not operating a transparent peer review scheme. This document only contains reviewer comments and rebuttal letters for versions considered at *Nature Communications*.

**Reviewer comments & decisions:**

**Reviewer comments, first version:**

Reviewer #1 (Remarks to the Author):

This is a significant piece of work which will be of great interest to atmospheric scientists, microplastic (MP) scientists and anthropic pollutant scientists. It is well written and a very substantial piece of work and will potentially be used as the new standard for atmospheric particle dispersion modelling of MP (FLEXPART modelling). This work will advance the potential understanding of tire wear MP impact on terrestrial and oceanic areas, the potential long-distance transport of TWP and BWP and push the MP modelling boundaries in the atmospheric environment.

The manuscript makes the following claims:
-That significant road MP is reaching that Arctic and other remote and snow/ice environments.
-That a significant proportion of atmospheric road MP is deposited on the oceans.
-That atmospheric transport of road MP is a significant vector and pathway for oceanic road MP, comparable and potentially greater in quantity than terrestrial runoff and river sourced oceanic road MP.
-Atmospheric road MP is underestimated as a global terrestrial and marine ecosystem threat.
-Arctic atmospheric road MP deposition may influence climate risk due to its affinity to absorb solar radiation and accelerate melting.
-Effective particle dispersion modelling of road MP by FLEXPART using novel but defended assumptions and parameterisations.

These claims, the modelling and the presented results are novel, well described and documented.

There are several atmospheric MP papers that provide reviews of the current science that are missing from this manuscript. These papers could be included in the manuscript for completeness and conciseness in up-to-date literature consideration. These are:
Enyoh et al. Airborne microplastics: a review study on method for analysis, occurrence, movement and risks. Environmental Monitoring and Assessment. 191, 1-17 (2019)
Zhang et al. Atmospheric microplastics: A review on current status and perspectives. Earth-Science Reviews. 203, 103118 (2020)
Chen, G., Feng, J. & Wang, J. Mini-review of microplastics in the atmosphere and their risks to humans. Science of the Total Environment. 703, 135504 (2020)
There are also recent papers on Antarctic Ice MP that could be included and discussed (further mentioned in the Detailed Comments - attached document).

Further clarification, discussion and, in places, additional or modified references are need on: the creation mechanisms of TWP and BWP, CCN, the use of the two different emission models (in the main text) and definition of road microplastics (please see the Detailed Comments). It should be clearly stated in the road microplastic definition that TWP and BWP are not the only types of MP from roads. It also needs to be made clear in the title and introductory text that this paper is focused on TWP and BWP specifically, not all road MP or all atmospheric MP.

Some additional clarification and review of the tables and value reporting is needed (comments in further detail in the Detailed Comments) and supplementary information on the code (FLEXPART) and the delineation of the discussed areas (Arctic, Europe, Asia etc) is needed to support future replication and comparable modelling and analysis.

I would recommend the authors undertake the recommended revisions and resubmit to this journal. This is a very promising manuscript.


Reviewer #2 (Remarks to the Author):

The manuscript describes the a modeling approach for emission and transport of tire wear and break wear particles in the atmosphere using different sources for their calculations and different scenarios while their results were appropriately supported by graphs and videos which made this complex modeling results well to understand. Still, important details for the modeling approach remain unclear for the reader which need to be addressed prior to further consideration.

General remarks: Why did the authors limit their discussion only on sea based areas and do not include remote land based areas suggested by the title? These should also be included.

Line 260 to 270: For BWP it is not clear how for 100% of the total BWP emission for BWP still up to 30 % can be emitted? In the current stage it can be read that up to 170 % and at least 130% of total BWP could have been used in the models. This is very confusing and the exact modeling procedure should be described in more detail as it is essential for this study and its reproducibility. In addition, please provide a table presenting the various model inputs, which allows the reader to exactly follow the procedure the authors applied.

Specific remarks:
Line 42 to 43: The author state that tires consist of elastomers and rubbers and different additives. This is very simplistic as they typically are designed/constructed during production and vulcanization around supports of e.g. polyamide fibers and other materials, which could also be emitted during use. Further, they also contain carbon black and silicates as filler materials to reduce the amount of elastomers used and to improve their stability. Please included a more accurate description of tires as they are a much more complex system.
Line 67 to 68: Please describe the lower size limit of the study here to avoid confusion as the current definitions are still debated (see e.g. Hartmann et al. 2019).
Line 91 to 92: Which is the annual and the daily mean? Is it respectively?
Line 203 to 205: The study of Bergmann et al. found microplastics with a majority of particles sizes smaller 25 µm, but fibers were significantly longer (see Figure S1 of the reference). Please amend the discussion below. Still, the authors should check if particles (these were 80% < 25 µm as also stated in line 201) as still fitting for their discussion and should include/investigate more work on Arctic Sea ice and surface waters e.g. the cited reference 6, Kanhai et al. 2018, 2020, Obbard et al. 2014.
Line 231. to 240: Please state a short comment in the results while introducing the modeled ranges as this was not clear at this early stage of the manuscript .
Materials and Methods: The authors used size classes for the different materials, but it is undefined how the size was linked to the mass. This should be shortly explained to avoid confusion.

Hartmann, N. B. et al. Are We Speaking the Same Language? Recommendations for a Definition and Categorization Framework for Plastic Debris. Environ Sci Technol 53, 1039-1047, doi:10.1021/acs.est.8b05297 (2019).
Kanhai, L. K. et al. Microplastics in sub-surface waters of the Arctic Central Basin. Mar. Pollut. Bull. 130, 8-18, doi:10.1016/j.marpolbul.2018.03.011 (2018).
Obbard, R. W. et al. Global warming releases microplastic legacy frozen in Arctic Sea ice. Earths Future 2, 315-320, doi:10.1002/2014ef000240 (2014).
Kanhai, L. D. K., Gardfeldt, K., Krumpen, T., Thompson, R. C. & O’Connor, I. Microplastics in sea ice and seawater beneath ice floes from the Arctic Ocean. Sci. Rep. 10, 5004, doi:10.1038/s41598-020-61948-6 (2020).


Reviewer #3 (Remarks to the Author):

Dear Authors

congratulations with an interesting and novel study. I enjoyed reading your paper and I have only a few suggestions that I believe would further improve your presentation and increase the value of your results.

1. The term "microplastics" usually addresses fragmented or virgin pieces of plastic polymers smaller than X centimeters, where X ranges between 0.5 and 5 cm.

TWPs and BWPs that you model are essentially not plastic in the common sense. These particles are also so small that they may be closer to nanoplastics.

To avoid confusing and potentially misleading terminology I suggest to use instead something like "synthetic (or artificial) microparticles".

2. As the authors explicitly stated, observational data that could be used to validate their simulations are currently not available.

However, it may be posible to use the data of other particulate matter (such as fibers or dust) whose dynamics while transported by the wind is similar.

Also the author could suggest sites or strategy of observations that would efficiently validate their model experiments.

**Author rebuttal, first version:**

Reviewer #1 (Remarks to the Author):

This is a significant piece of work which will be of great interest to atmospheric scientists, microplastic (MP) scientists and anthropic pollutant scientists. It is well written and a very substantial piece of work and will potentially be used as the new standard for atmospheric particle dispersion modelling of MP (FLEXPART modelling). This work will advance the potential understanding of tire wear MP impact on terrestrial and oceanic areas, the potential long-distance transport of TWP and BWP and push the MP modelling boundaries in the atmospheric environment.

The manuscript makes the following claims:
-That significant road MP is reaching that Arctic and other remote and snow/ice environments.
-That a significant proportion of atmospheric road MP is deposited on the oceans. 
-That atmospheric transport of road MP is a significant vector and pathway for oceanic road MP, comparable and potentially greater in quantity than terrestrial runoff and river sourced oceanic road MP.
-Atmospheric road MP is underestimated as a global terrestrial and marine ecosystem threat.
-Arctic atmospheric road MP deposition may influence climate risk due to its affinity to absorb solar radiation and accelerate melting.
-Effective particle dispersion modelling of road MP by FLEXPART using novel but defended assumptions and parameterisations.

These claims, the modelling and the presented results are novel, well described and documented.

**Response**: We appreciate Reviewer 1 for very constructive comments, which will certainly improve our manuscript.

There are several atmospheric MP papers that provide reviews of the current science that are missing from this manuscript. These papers could be included in the manuscript for completeness and conciseness in up-to-date literature consideration. These are:
Enyoh et al. Airborne microplastics: a review study on method for analysis, occurrence, movement and risks. Environmental Monitoring and Assessment. 191, 1-17 (2019)
Zhang et al. Atmospheric microplastics: A review on current status and perspectives. Earth-Science Reviews. 203, 103118 (2020)
Chen, G., Feng, J. & Wang, J. Mini-review of microplastics in the atmosphere and their risks to humans. Science of the Total Environment. 703, 135504 (2020)
There are also recent papers on Antarctic Ice MP that could be included and discussed (further mentioned in the Detailed Comments - attached document).
**Response**: We have included these articles in the manuscript.

We hope that the journal will allow us to use so many references, considering that the present paper is not a review paper.

Further clarification, discussion and, in places, additional or modified references are need on: the creation mechanisms of TWP and BWP, CCN, the use of the two different emission models (in the main text) and definition of road microplastics (please see the Detailed Comments). It should be clearly stated in the road microplastic definition that TWP and BWP are not the only types of MP from roads. It also needs to be made clear in the title and introductory text that this paper is focused on TWP and BWP specifically, not all road MP or all atmospheric MP.

**Response**: We have largely followed the reviewer’s comments. Please see manuscript with track changes.

Some additional clarification and review of the tables and value reporting is needed (comments in further detail in the Detailed Comments) and supplementary information on the code (FLEXPART) and the delineation of the discussed areas (Arctic, Europe, Asia etc) is needed to support future replication and comparable modelling and analysis.
**Response**: We have followed reviewer’s comments. Please see manuscript with track changes. We have also added a supplementary figure (Fig. S3) with the masked areas and uploaded the files that we used on DRYAD.

I would recommend the authors undertake the recommended revisions and resubmit to this journal. This is a very promising manuscript.

**Response**: We have followed reviewer’s comments. Please see manuscript with track changes.

Comments in detail

Title: the title is not specific enough with regards to the topic. The title needs to include

Tire Wear and Brake Wear to ensure the reader knows exactly what the manuscript topic

is. This paper does not discuss any other MP type in detail, only tire and brake particles.

This must be made clear in the title.

**Response**: In principle, the reviewer is right here, and we have sharpened our definition of “road microplastics” in the paper. However, we would nonetheless prefer to keep the current title. Tire Wear (TWPs) and Brake Wear Particles (BWPs) are some of the largest (outdoor) sources of microplastics. Although many other sources of microplastics could be included, we examine only TWPs and BWPs, simply because we know their global sources reasonably well. Some of our results (e.g., transport efficiencies) likely apply to other sources of microplastics as well if the emitted particles are in the same size range as considered here. Therefore, the results of the paper should be of interest also for readers studying other types of microplastics.

Besides, most of the current research that examines sources or environmental dispersion of microplastics refers to specific types; yet, these particles are characterised as microplastics (e.g., Bergmann et al., 2019, where fibres are also included, but all described as microplastics in the title; Lusher et al., 2015, where fibres, fragments and films were examined and all called microplastics in the title; and many others).

In addition, Hartmann et al. (2019) have tried to specify a common terminology to be used for scientific reason. They use the term “microplastics” only based on the particle size, exactly as we have done in the present paper.

Bergmann, M. et al. White and wonderful? Microplastics prevail in snow from the Alps to the Arctic. Sci. Adv. 5, 1–11 (2019).

Hartmann, N. B. et al. Are We Speaking the Same Language? Recommendations for a Definition and Categorization Framework for Plastic Debris. Environ Sci Technol 53, 1039-1047, doi:10.1021/acs.est.8b05297 (2019).

Lusher, A. et al. Microplastics in Arctic polar waters: the first reported values of particles in surface and sub-surface samples. Sci Rep 5, 14947 (2015). https://doi.org/10.1038/srep14947

Page 2, line 19: ‘as a major source’. Please add ‘of atmospheric pollutants’ or an

appropriate descriptor here for clarity and flow.

If this is a major MP source, this needs to be justified through appropriate reference

considering all MP types and identified and quantified emission sources. Please provide

appropriate references to support this statement.

**Response**: We have added references in page 2 line 8, instead. In most of the scientific journals, references are not allowed in the abstract, because abstract reflects what is written in the main body of the manuscript.

Page 1, line 28: Please be specific and clear. Brake and tire wear are not the only sources

of road related microplastic. Therefore, it is not accurate to use the term ‘road

microplastics’ here as it suggests you have considered all road MP, not just tire and brake

wear. Please only use the term ‘road microplastics’ after you have clarified and described

this term in the main text.

**Response**: Done as suggested. We now use “road microplastics” only after we have defined the term for our purposes!

Page 2, Line 32: Update the global production to 2018 values, along with the appropriate

reference. PlasticsEurope has published the 2018 values (in Plastics, the facts 2019) and

these are greater than 348 million tonnes. Please update this to the most current

figures.

**Response**: Corrected!

Page 3, Line 47: Brakes are comprised of a disc or drum with either a âir of shoes or pads

mounted in calipers. Please amend for clarity.

**Response**: Corrected!

Page 3, Line 55: Please check the use of the terms adsorbing and absorbing

**Response**: Corrected!

We appreciate reviewer’s help that grabbed this tiny detail here. As it is described in the Discussions section of the following paper, adsorption is the correct terminology here.

Ashton, K., Holmes, L. & Turner, A. Association of metals with plastic production pellets in the marine environment. Mar. Pollut. Bull. 60, 2050–2055 (2010).

Page 3, Line 56: ‘Larger’ should be ‘Greater’ or ‘More intensive use of plastics…’

**Response**: Corrected!

Page 3, Line 58: ‘…long remain airborne’ is difficult to read and breaks the readers flow.

Please amend to ‘’they can remain airborne for long or extended periods of time…’ or

similar.

**Response**: Corrected!

Page 3, Line 59: Not sure that TWP or BWP have been proven to absorb light and affect

ice/snow albedo. It is possible, but the cited paper discusses black carbon. Please add

black MP citations and/or clarify the discussion relative to known research findings (i.e.

BC rather than MP).

**Response**: Corrected!

Page 4, line 61: Nature journals generally do not support the use of phrases such as ‘for

the first time’. It is recommended that you please remove this text to comply with

Nature’s recommendations. If it is published in NComms, it is novel and it is not

necessary to state that to the readers.

**Response**: Corrected!

Page 4, Line 63: ‘one indirect and another using an emission model’. This statement is

difficult to read or understand. While the details are provided in the methods, and the

authors note this in the (see Methods) comment, can this sentence please be reworded

and possibly expanded to provide clarity and information to the reader on the

approaches?

**Response**: Corrected!

We understand that this statement stops the flow of reading and we have removed it. We think it is enough that we point to the methods section, where we explain in detail the 2 methodologies.

Page 5, Line 64: Please include an explaination of why only BWP emission were

calculated sith the emission model. It is acknowledged this is discussed in the lethids, but

an explainatory statement is also needed here.

**Response**: Sentence removed!

We explain this in the Methods section and we believe we do not need to provide these details here.

Page 4, Line 61-68: To improve the flow of the manuscript and minimise repetition, it is

recommended this paragraph be removed (deleted) in its entirety.

**Response**: This paragraph shows what we intend to present in this paper and we think this is necessary. We would prefer to follow reviewer’s detailed comments and try to improve it rather than to remove it.

Page 4, Line 64-65: tire and brake wear are not the only microplastics that come from or

are related to traffic and road microplastics. Can you please include a statement

acknowledging this prior to or just after defining your use of the term ‘road

microplastics’. This is necessary for clarity in this discussion of tire and brake MP.

**Response**: Corrected! We also give examples for other road traffic sources not considered here.

Page 4, Line 67-68: ‘…long remain airborne’ is difficult to read and breaks the readers

flow. Please amend to ‘’they can remain airborne for long or extended periods of time…’

or similar.

**Response**: Corrected!

Page 4 Line 71: Please include the reference for the CO2 ratio method here (56?) for

clarity.

**Response**: Corrected!

In fact one of the most highly cited papers on biomass burning emissions (Andreae and Merlet, 2001) has used this method and we have added this reference here.

Andreae, M. O. & Merlet, P. Emissions of trace gases and aerosols from biomass burning. Global Biogeochem. Cycles 15, 955–966 (2001).

Page 4 Line 72: Please include the reference for the GAINS model method here (62?) for

clarity.

**Response**: Corrected!

Page 4, Line 73: For clarity, please state in full what kt is the first time you use it.

**Response**: Corrected!

Page 4, Line 73: Two values are presented here, 3434 kt and 2380 kt, and state the

results from these two methods have been averaged for use in the modelling activities.

This section would benefit from further description or discussion to illustrate that the

authors have completed both a sensitivity and differences analysis (Extended data Fig 1.)

and that this sensitivity and uncertainty ranges between 2.1-3 (geometric standard

deviation) and 0.25-32 t/yr in difference (range of difference between the two emission

methods) for tire wear, and 1-1.2 (?) geometric standard deviation for brake wear. For

clarity, it is recommended this is simply stated in a sentence or two here before going

into detail on the emissions by location in the next paragraph.

**Response**: Corrected! We now explain in more detail.

Two emission methods were used for tire wear but only one for brake wear emissions.

Please provide a simple explanation in the methods on why brake wear was estimated

using only one method (GAINS) for clarity.

**Response**:

We have added a small paragraph in Methods section explaining that for tire wear we can use information on the weight of returned tires to estimate the emissions. This information is not available for breaks, so break wear cannot be estimated that way.

Also for clarity, in the methods section can you provide a map delineating clearly the

geographical extents of each of your continental partitions (Europe, Asia without Russia,

Russia, North America, Central America, South America etc…). While this potentially a

general knowledge issue, this paper may be used as the standard for future work and

clarity in detail is therefore important.

**Response**: Supplementary Figure S3 has been added, where all reference regions are plotted. All files have been uploaded on DRYAD and are publicly available.

Extended data figure and tables: Extended data table 1 and data figure 1 has errors in its

referencing requiring amendment.

**Response**: Corrected!

Extended data table 1 has now become Extended data table 2 and links have been updated.

Page 4 Line 78-79: Please clarify how the values of TWP and BWP emission were

calculated – 2907 kt/yr and 175kt/yr (specifically the value differences between Fig 1

‘Annual emission of total TWP and BWP’ and Table 1). Please provide a reference or link

in the paper to the appropriate table that presents these values (e.g. Fig1 panel a and b).

Ideally this total value should be relatable directly with the figures in Table 1, so if

possible include these values in Table 1 to create consistency in reporting.

**Response**: Corrected! We further explain this in page 4 providing links to Fig. 1.

Table 1. Please check the summation in this table to ensure readers can use these values

in future analysis correctly. Please also ensure the decimal place use in consistent across

the table (e.g., totals of 29.5, 228.2, 98.0, 146.0). It is also recommended this review is

undertaken for Table 2 values as well, and if truncation is used rather than rounding for

this to be stated in the Table legend or as a note.

**Response**: A large effort has been made to present values in the tables correctly prior to submission of this article. We always keep the same number of significant digits in the ranges and in the geometric mean (given in parentheses). We have only found one mistake in Table 2, which we have corrected.

For the totals, when the range of total PM2.5 BWP is 63.4 – 152, we present 3 significant digits, and then the geometric mean should have 3 digits as well (98.2). However, for PM2.5 TWP that smaller emissions have been calculated, the range should have 2 significant digits, 12 – 75 (29).

We look forward for further comments on this, if the reviewer insists that the presentation is inconsistent. Below, we kindly indicate a book that we have followed, which shows how to present scientific results:

<https://doi.org/10.1007/978-3-319-53345-2>

Page 4 Line 86: suggested amendment ‘highest in Asia (Table 1, Extended data Fig 2).’ Or

state the correct highest BWP PM2.5 location. It is also noted that this nuance in results

is not easily visible in Extended Data Fig 2. due to the capping of colour trends at 32. If it

is possible to amend this figure to illustrate the Asian continental values of 58 (50-67) kt/

yr? This would be beneficial to the reader. This comment is also made for the PM10 BWP

and both the TWP extended data figure 1. It is noted that the top value of 32 is used for

consistency in all these figures, but it would be useful to have colour scales specific to

the PM size and particle type (TWP or BWP) just because there is some much detail and

variation in the results and this could be really useful in digesting the findings of this

work.

**Response**: At first, the reason that we have used a log2 scale in the colorbar is exactly this; to capture any regional spatial details on the maps, which we believe we do, since the total difference between PM2.5 and PM10 emissions of BWP are less than a factor of 2. As for the TWP, Extended Data Fig. 1 has 8 panels and using 8 different colorbars would shrink the maps a lot, making any spatial difference practically invisible. So, we do not really know if any change would result in something beneficial or would complicate things here.

The value of 58 (50-67) kt/yr that the reviewer mentions here is the total emissions in the Asian continent as defined in the masks that we have provided with this paper on DRYAD and are now included in the revised version of the Supplements. It is also explained in the caption of the table that the values correspond to continental emissions.

Page 5, Line 91: Southeastern should be hyphenated to South-eastern

**Response**: Corrected everywhere in the text!

Page 5, Line 94: amend to ‘smaller road microplastic particles’ to ensure consistency

**Response**: Corrected!

Page 5, Line 94-96: recommend revising the first sentence to form 2 more succinct

sentences. e.g. Annual deposition maps show that smaller road microplastic particles

(PM2.5) are dispersed more widely than larger ones (PM10). PM10 road microplastic

particles predominantly deposited close to the hotspot emission regions (North

America, Europe and Southeastern Asia).

**Response**: Corrected!

Page 5, Line 103: Please add a note in the methods section on the definition of

‘mountains etc.’ (elevation, snow cover for x% of the year…) so that this analysis could

potentially be replicated by future researchers.

**Response**: Corrected!

We anyway provide all datasets through DRYAD, for anyone that needs to replicate these findings. We have added a note in Supplementary Information and in Data Availability.

Page 5, Line 104: the % values provided for TWP PM2.5 are definitive, but the TWP PM10

is approximate (~). Why? If possible, please provide a definitive modelled output value

here.

**Response**: Corrected!

Since we give ranges of the mass deposited in any region, we think it is better to use approximate values everywhere, in order to be consistent and avoid the use of ranges when discussing percentages in this chapter.

Page 5, Line 106: Please amend ‘Transport’ to ‘Deposition’ for accuracy.

**Response**: Corrected!

Page 5, Line 106 and 115: Negligible TWP or BWP was found to deposit in Antarctica. It

would be beneficial to include a comment on Antarctic ice core MP reported (e.g. Kelly et

al. 2020 doi: 10.1016/j.marpolbul.2020.111130) noting that while TWP or BWP not

modelled to occur in the Antarctic region, other forms of MP have been found there and

may have been transported via sea or air.

**Response**: Corrected!

Page 5, Line 119: Amend to ‘Concentrations of TWP and BWP in snow-covered…’ for

accuracy as there are many other types of MP beyond brake and tire wear.

**Response**: Corrected!

To be consistent with a previous comment from the same reviewer, we have changed the title to “Concentrations of road microplastics in snow-covered land and ice surfaces”, which agrees with the definition we have given in the last paragraph of the introduction section.

Page 6, Line 125: ‘ present higher TWP/BWP snow concentrations…’ amendment for

clarity and consistency

**Response**: Corrected!

Page 6, Line 131-132: North Atlantic Oscillation comment on transport is interesting and

it would be beneficial if this could be expanded a little to explain further to discuss what

the influence of the NAO may be (e.g. strongest positive NOA occurring in Feb 2014

(positive during Jan-March and Nov-Dec) leading to a high pressure shift that potentially

supports greater entrainment and atmospheric transport of tire MP from xx continental

areas up to xx arctic locations through wind, dry and snow deposition etc…..) and to

include comment on the potential Arctic Oscillation influence.

**Response**: We have added a few sentences on the NAO’s impact! We now write:

Transport of microplastics into the Arctic occurs particularly in winter and spring (Fig. 3e-f, Fig. S 1) and is likely enhanced during positive phases of the North Atlantic Oscillation (NAO)^58^. It is known from previous work^58^ that pollution transport from North America and Europe is enhanced during positive phases of the NAO and that this effect is strongest in winter and spring. Since 1990, there have been seven years with high NAO indexes, a negative phase around 2010 and again positive phase between 2014 and 2019^59^.

However, without explicit model calculations we cannot give concrete numbers for microplastics.

Page 6, Line 142-144: This sentence suggests that only PM10 sized microplastic can

become airborne. Given that there are numerous atmospheric microplastic studies now

published with deposited or airmass microplastics of greater than 10μm, the PM10

inclusion in this sentence is not accurate. Please amend, removing the suggested PM10

limitation of atmospheric MP size. I think you were trying to relate to this studies

particle size limit, but the text does not read this way and needs revision.

**Response**: Corrected! We have removed the suggested PM10 limitation

Page 6, Line 145: … decreases while vehicle speed decreases (please amend to be

accurace)

**Response**: Corrected!

Page 6, Line 143-144 ‘PM10 microplastic deposition’ needs to be amended to road

microplastic deposition within the PM10 range, or road microplastics ≤10μm. Please

make sure road, BWP or TWP wording is used throughout the manuscript when

discussion MP to ensure accuracy in your discussion and not MP generalisation unless

this is the specific intention of the comment.

**Response**: Corrected!

We have now clearly defined at the beginning what we mean with “road microplastics”, so it should be clear to the reader that only TWP and BWP are included in this study.

Page 7, Line 152-154: This states that gravitational settling and below-cloud scavenging

of larger particles is more important for PM10 sized road MP compared to PM2.5. This

statement requires further discussion to explain why this is (e.g. the action of belowcloud

scavenging on road MP of PM10 size range relative to PM2.5 etc…), with

appropriate references.

**Response**: Corrected!

The statement “less efficiently deposited there” is not accurate. We have changed to “less efficiently transported there”, because we intend to highlight that this is done due to gravitational settling differences of PM10 vs PM2.5 size particles, as it is also explained in the next sentence.

Page 7, Line 170: The manuscript states there is negligible deposition on the Southern

Ocean. Compared to the Atlantic, Arctic, Indian etc. This is accurate according to the

model output, however compared to the Alps, Himalayas, Baltic Sea etc. this statement

seems less accurate. Please revise this statement for accuracy.

**Response**: We now also provide concrete numbers for the Southern Ocean.

Page 7, Line 172: ‘road microplastic emissions’

**Response**: Corrected!

Page 7, Line 178: This statement is inaccurate. The word ‘important’ should not be used

here as this manuscript does not demonstrate or discuss the importance of MP

atmospheric deposition but presented to modelled occurrence and concentration or

road MP deposition. Please amend this to ‘…where deposition of small particles is

greater than PM10 road MP…’ or similar.

**Response**: Amended as suggested!

Page 9, Line 1: the climate risk of MP polution has not yet been proven, so it is more

accurate to state these transport patterns may potentially intensify the climate risk,

rather than using the definitive wording.

**Response**: Corrected!

We completely agree with this statement and we have changed it following reviewer’s suggestion.

Page 8 Line 198-200: Bergmann et al. 2019 only analysed down to 11μm due to the

limitations in analytical methodology (μFTIR). While it is noted in the manuscript that

most particles were <25μm, the accurate statement should be most particles identified

were between 11-25μm with potentially <11μm but that these were not counted or

identified due to methodological limitations.

**Response**: We agree and have corrected!

We appreciate reviewer’s persistence to improve our paper.

Page 9, Line 219 This is a very strong statement. The ‘importance’ of TWP atmospheric

deposition on oceans discussed here relates to quantity, but could potentially be missread

as environmental or ecological importance (relating to impact). Please revise this

statement to highlight that airborne road MP could be a significant and important

source of ocean road MP, with clarity in the wording to ensure impact (env/ecol) is not

discussed (or alternatively provided in the discussion in significant detail).

**Response**: Corrected!

We admit that when talking about marine biota, one may misuse it to imply impact; therefore, we have removed it, in order to be more straight-forward in our statement. Now, it has changed as follows: “This suggests that direct deposition of airborne road microplastics is likely the most important source for the ocean”.

Methods

Method comment on road microplastic particle shape is needed. A clear and detailed

discussion on the particle size is provided in the methods section and comment on

particle densities. Furthermore, clear assumptions supported by references on in and

below cloud scavenging are provided in Extended data Table 1. However, the

microplastic community is still trying to address the shape issue with regards to

transport – in all medium (air, water, soil etc.) and to support future consistency in

research and for clarity a comment on the assumptions of shape of the TWP and BWP

both PM2.5 and PM10 (as modelled and considered in this study) is needed in this

methods section.

**Response**: Corrected!

Very good comment that helps reader to understand how MP particles were modelled in FLEXPART! We have added a sentence in the beginning of the second paragraph in Methods (section where the FLEXPART description is given), as follows: “FLEXPART assumes a spherical shape of particles^79^ and, as such, the dispersion of road microplastics was modelled.”

Page 9, Line 228 and Page 10, Line 252: In the first instance 2014 is mentioned as the

year used to create representative CO2 ratio TWP values, but GAINS method calculations

for TWP and BWP uses 2015 values. Is this an error or where two different years used to

create representative data for this modelling study? If different years were used, please

explain why and the potential impact this may have on the comparability of CO2 ratio

and GAINS method emission calculations used here and the impact of this on the relative

modelling time period (2014).

**Response**: Emissions from the GAINS model are only produced in 5-year periods (see Klimont et al., 2017, <https://doi.org/10.5194/acp-17-8681-2017>). On the other hand, statistical data from returned tires for Norway, Sweden and Germany were only available for year 2014 and these could only be used in the CO2 method.

We think that having emissions from two independent methods gives more representative estimates on real emissions of TWPs. The fact that these emissions refer to two consecutive (different) years and how this would affect the resulting emissions cannot be assessed. Though, considering that the GAINS model runs every 5 years to record global emission differences (see also <https://iiasa.ac.at/web/home/research/researchPrograms/air/ECLIPSEv5a.html>), we do not expect large bias.

Page 11, Line 283: evaporation and re-condensation of TWP and BWP? This sentence is

dificult to read and suggests the road MP are being evaporated and re-condensed.

Please revise for clarity for the reader.

**Response**: Corrected!

Page 11, Line 294-297: Not sure that reference on rubber in cement is the best one to

describe CCN properties. The hydrophobic nature of TWP may not have a large effect on

its CCN properties as much as hygroscopic nuclei. Large particle presence can often be

enough regardless of surface charge. Ganguly et al. Paper on MP/NP ice nucleation

migth be helpful, <https://doi.org/10.1021/acsearthspacechem.9b00132>

**Response**: This very helpful reference has been included!

Page 12, Line 303: ‘…simulations also accounted for below-cloud scavenging and dry

deposition…’ amendment.

**Response**: Corrected!

Page 12, Line 312: ‘…eight that are in the …’ amendment or similar for grammatical

Accuracy

**Response**: Corrected!

Page 12, Line 313: ‘…three that are in the …’ amendment or similar for grammatical

Accuracy

**Response**: Corrected!

Reviewer #2 (Remarks to the Author):

The manuscript describes a modeling approach for emission and transport of tire wear and break wear particles in the atmosphere using different sources for their calculations and different scenarios while their results were appropriately supported by graphs and videos which made this complex modeling results well to understand. Still, important details for the modeling approach remain unclear for the reader which need to be addressed prior to further consideration.

**Response**: We appreciate the reviewer’s willingness to clarify confusing parts of our paper.

General remarks: Why did the authors limit their discussion only on sea based areas and do not include remote land based areas suggested by the title? These should also be included.

**Response**: Table 2 shows deposition of TWPs and BWPs in Europe, Asia, Russia, N America, C. America, South America, Africa, Oceania, Antarctica, whereas Table 3 shows transport efficiencies (fraction of emissions that was deposited over a certain region) for the Arctic, Alps, Himalayas, Greenland, all Oceans, Mediterranean Sea, Baltic Sea and South China Sea.

This shows that we include all the relevant information of microplastic transport to not only oceanic, but also continental regions. One can understand that we cannot discuss all the computed numbers in detail in the main text due to the journal size limitations, but the information is in the tables.

Line 260 to 270: For BWP it is not clear how for 100% of the total BWP emission for BWP still up to 30 % can be emitted? In the current stage it can be read that up to 170 % and at least 130% of total BWP could have been used in the models. This is very confusing and the exact modeling procedure should be described in more detail as it is essential for this study and its reproducibility. In addition, please provide a table presenting the various model inputs, which allows the reader to exactly follow the procedure the authors applied.

**Response**: We acknowledge reviewer’s help to improve this part and admit it is confusing. We have added Extended Data Table 1 in an effort to make it clearer.

From this comment, we understand that the reviewer has summed PM2.5 and PM10. This is fundamentally incorrect, because PM2.5 is always part of PM10, and PM10 must -by definition- always be more than PM2.5 (assuming that there are some particles larger than 2.5 um); thus they should not be summed. We believe we have made this clear now, but we further give some more examples below.

According to EPA (1995), the emission factor (EF) of TSM BWP for cars and trucks is 0.0080 g/km, the PM2.5 BWP EF is 0.0037 g/km and PM10 BWP EF is 0.0078 g/km. So, for 1000 km driven we would get 8 g of total BWP emissions, of which 3.7 g are PM2.5 BWPs and 7.8 g are PM10 BWPs.

According to the RAINS model, the EFs for light-vehicle cars are 0.0022 g/km for PM2.5 BWPs, 0.0014 g/km for coarse BWPs, 0.0036 g/km for PM10 BWPs, 0.0008 g/km for >PM10 BWPs and 0.0044 g/km for TSM BWPs. For 1000 km driven, we would have 2.2 g of PM2.5 BWPs, 1.4 g of coarse BWPs, 3.6 g of PM10 BWPs, 0.8 g of >PM10 BWPs and 4.4 g of TSM BWPs.

EPA (Environmental Protection Agency) (1995) Compilation of air pollution emission factors,Vol.1 and Vol.2, AP-42, 5th edition.

Klimont, Z. et al. Modelling Particulate Emissions in Europe. IIASA, Interim Rep. IR-02-076, (2002).

Specific remarks:

Line 42 to 43: The author state that tires consist of elastomers and rubbers and different additives. This is very simplistic as they typically are designed/constructed during production and vulcanization around supports of e.g. polyamide fibers and other materials, which could also be emitted during use. Further, they also contain carbon black and silicates as filler materials to reduce the amount of elastomers used and to improve their stability. Please included a more accurate description of tires as they are a much more complex system.

**Response**: We admit that we had simplified the introduction and thus the description of the composition of tires, due to the size limitations of the journal. We have now extended it as the reviewer suggested (see track changes version of the manuscript).

Line 67 to 68: Please describe the lower size limit of the study here to avoid confusion as the current definitions are still debated (see e.g. Hartmann et al. 2019).

**Response**: We appreciate reviewer’s help to further clarify our definitions. We have now added the specific particle sizes examined in the present paper and have cited Hartmann et al. paper, which we believe is very useful to support our definitions.

Line 91 to 92: Which is the annual and the daily mean? Is it respectively?

**Response**: As we explained in “Atmospheric transport modelling of road microplastics” sub-section of Methods section (last sentence), the temporal resolution in our model was set as such to give daily mean outputs. Regarding annual mean values, we have now added the value in parenthesis.

Line 203 to 205: The study of Bergmann et al. found microplastics with a majority of particles sizes smaller 25 µm, but fibers were significantly longer (see Figure S1 of the reference). Please amend the discussion below. Still, the authors should check if particles (these were 80% < 25 µm as also stated in line 201) as still fitting for their discussion and should include/investigate more work on Arctic Sea ice and surface waters e.g. the cited reference 6, Kanhai et al. 2018, 2020, Obbard et al. 2014.

**Response**: Kanhai et al. (2020) determined size class distributions of microplastics in the ice cores of: 0.1–0.5 mm (32%), 0.5–1 mm (20%), 1–2 mm (23%), 2–3 mm (13%), 3–4 mm (7%), 4–5 mm (4%) (see page 2 of the original manuscript. Kanhai et al. (2018) detected underwater microplastics along the Central Arctic of sizes 0.25-5 mm (see Fig.4 of the original article). Finally, Obbard et al. (2014) determined microplastics of sizes >0.02 mm in Arctic ice-cores with FTIR (although they used 0.22 μm Millipore nitrocellulose filters, see methods section).

In the present study, we examine particles of mean size 0.5–9.5 µm or at least 20 times smaller. Although the aforementioned research articles provide evidence that microplastics have been recorded in Arctic regions, comparisons with our results are not possible because of the rather large size differences.

Though, we have included the references to provide additional evidence of microplastics’ presence in remote regions.

Line 231. to 240: Please state a short comment in the results while introducing the modeled ranges as this was not clear at this early stage of the manuscript .

**Response**: We do not understand what kind of comment is needed here, as it is not specified what was unclear in the reviewer’s comment. We could certainly correct for this comment in a next review.

Materials and Methods: The authors used size classes for the different materials, but it is undefined how the size was linked to the mass. This should be shortly explained to avoid confusion.

**Response**: The mass fraction that we used in the size distribution is clearly shown in Extended Data Figure 3 for TWPs and BWPs, respectively. We have now made it clear in Methods section and we point to the Extended Data Figure 3. They are shown in page 11-12.

The assumed size and mass are directly related through particle density and the use of spherical particle shapes.

Hartmann, N. B. et al. Are We Speaking the Same Language? Recommendations for a Definition and Categorization Framework for Plastic Debris. Environ Sci Technol 53, 1039-1047, doi:10.1021/acs.est.8b05297 (2019).

Kanhai, L. K. et al. Microplastics in sub-surface waters of the Arctic Central Basin. Mar. Pollut. Bull. 130, 8-18, doi:10.1016/j.marpolbul.2018.03.011 (2018).

Obbard, R. W. et al. Global warming releases microplastic legacy frozen in Arctic Sea ice. Earths Future 2, 315-320, doi:10.1002/2014ef000240 (2014).

Kanhai, L. D. K., Gardfeldt, K., Krumpen, T., Thompson, R. C. & O’Connor, I. Microplastics in sea ice and seawater beneath ice floes from the Arctic Ocean. Sci. Rep. 10, 5004, doi:10.1038/s41598-020-61948-6 (2020).

Reviewer #3 (Remarks to the Author):

Dear Authors

congratulations with an interesting and novel study. I enjoyed reading your paper and I have only a few suggestions that I believe would further improve your presentation and increase the value of your results.

**Response**: We would like to thank Reviewer 3 for these kind comments.

1. The term "microplastics" usually addresses fragmented or virgin pieces of plastic polymers smaller than X centimeters, where X ranges between 0.5 and 5 cm.
TWPs and BWPs that you model are essentially not plastic in the common sense. These particles are also so small that they may be closer to nanoplastics.
To avoid confusing and potentially misleading terminology I suggest to use instead something like "synthetic (or artificial) microparticles".

**Response**: The definition we borrowed from Peeken et al. defines microplastics as those particles of size between 1 μm to 5 mm. In the present paper we examined the dispersion of TWPs and BWPs in the PM2.5 and PM10 mode, hence, we fall in this definition. As regards to the characterization of these microplastics as “synthetic”, we would like to avoid using it. In numerous papers, the characterization “synthetic” is used along with microplastics to imply fibers from clothes (see for instance Jamieson et al., 2019; De Falco et al., 2018), which is not the case here.

But also the characterization “artificial” is not entirely true here, in our opinion. As we explain in the beginning of the 3^rd^ paragraph of our Introduction, tires consist of a mixture of elastomers such as rubber, which can be natural or synthetic/artificial, and different additives (Sundt et at., 2016).

Peeken, I. *et al.* Arctic sea ice is an important temporal sink and means of transport for microplastic. *Nat. Commun.* **9**, (2018).

A. J. Jamieson, L. S. R. Brooks, W. D. K. Reid, S. B. Piertney, B. E. Narayanaswamy and T. D. Linley 2019. Microplastics and synthetic particles ingested by deep-sea amphipods in six of the deepest marine ecosystems on Earth. R. Soc. open sci. 6:180667, <https://doi.org/10.1098/rsos.180667>

De Falco, F., PiaGullo, P., Gentile, G., Di Pace, E., Cocca, M., Gelabert, L., Brouta-Agnésa, M., Rovira, A., Escudero, R., Villalba, R., Mossotti, R., Montarsolo, A., Gavignano, S., Tonin, C., Avella, M. 2018. Evaluation of microplastic release caused by textile washing processes of synthetic fabrics, Environmental Pollution 236, 916-925, <https://doi.org/10.1016/j.envpol.2017.10.057>

Sundt, P., Syversen, F., Skogesal, O. & Schulze, P.-E. Primary microplastic-pollution: Measures and reduction potentials in Norway. Miljodirektoratet.No 117 (2016). Available at: <http://www.miljodirektoratet.no/Documents/publikasjoner/M545/M545.pdf>. (Accessed: 7th May 2020)

2. As the authors explicitly stated, observational data that could be used to validate their simulations are currently not available.
However, it may be posible to use the data of other particulate matter (such as fibers or dust) whose dynamics while transported by the wind is similar.

**Response**: The model has already been validated for dust particles (see Groot Zwaaftink et al., 2016; 2017). Fiber particles are not yet simulated in the model due to the assumption of spherical particles that is done within FLEXPART. We have started planning relevant development in order to be able to simulate such particles. In addition, we do not see how model validation using measurements of different atmospheric substances would help make a conclusion on the dispersion of microplastics in remote regions. If this is the case, then there are several hundreds of peer-reviewed papers dealing with comparison of FLEXPART with CO, BC, dust, N2O, radionuclides, CH4 etc. (see <https://folk.nilu.no/~andreas/publications/index.html>) .

Groot Zwaaftink, C.D. Ó. Arnalds, P. Dagsson-Waldhauserova, S. Eckhardt, J. M. Prospero, A. Stohl (2017), Temporal and spatial variability of Icelandic dust emission and atmospheric transport. Atmospheric Chemistry and Physics, 17, 10865-10878, doi:10.5194/acp-17-10865-2017.

Groot Zwaaftink, C. D., H. Grythe, H. Skov, A. Stohl (2016), Substantial contribution of northern high-latitude sources to mineral dust in the Arctic, Journal of Geophysical Research, 121, 13,678–13,697, DOI: 10.1002/2016JD025482.

Also the author could suggest sites or strategy of observations that would efficiently validate their model experiments.

**Response**: We have added a short sentence to suggest a proper strategy for measurements of microplastics (see last sentence of 6^th^ paragraph in Discussions).

**Reviewer comments, second version:**

Reviewer #1 (Remarks to the Author):

Thank you for the opportunity to review this work and for the efforts from the Authors to make adjustments and improvements to the manuscript.

The Authors have addressed all comments presented by the reviewer, and where necessary made effective changes to the manuscript. It is noted that the Authors do not want to provide any modification to the manuscript title. The reviewer still considers additional amendment to the title to be beneficial to the paper, especially as this is expected to be a highly cited and read piece of work. However, while the reviewer continues to recommend a title amendment ‘Atmospheric transport, a major pathway of tyre and brake wear microplastics to remote regions‘, the Authors have provided additional comment on their stance. Advice from the Editor on this is requested and their guidance accepted with regards to the manuscript title.

The reviewer acknowledges and understands the method of data analysis and presentation in the Tables (e.g. Table 1). Presentation in significant figures is one way to provide this data. However, it may still be easier for readers to read the information if it is provided to a defined decimal point value that is consistent (e.g. to 1 dp place). However, the reviewer acknowledges the use of both methods.

All other comments previously provided have been well addressed and the reviewer thanks the authors for the additional information, analysis and effort. The reviewer looks forward to reading the published version of this manuscript.


Reviewer #2 (Remarks to the Author):

The authors addressed all raised issues in an appropiate and detailed manner.


Reviewer #3 (Remarks to the Author):

I feel that the authors adequately and sufficiently addressed all questions, comments, and suggestions and that discussion can be continued after publication of the manuscript with the participation of a broader readership.

**Author rebuttal, second version:**

**Reviewer comments, third version:**

**Author rebuttal, third version:**
